# Genome-Wide Identification and Analysis of GATA Gene Family in *Dendrobium officinale* Under Methyl Jasmonate and Salt Stress

**DOI:** 10.3390/plants14111576

**Published:** 2025-05-22

**Authors:** Zhangting Xu, Feixiong Zheng, Xiaoji Deng, Yiming Sun, Zhenming Yu, Xiaoxia Shen

**Affiliations:** 1 School of Pharmaceutical Sciences, Zhejiang Engineering Research Center for Chinese Medicinal Plant Essential Oils, Zhejiang Chinese Medical University, Hangzhou 310053, China; 15168104031@163.com (Z.X.); feixiongz@163.com (F.Z.); 13868867263@163.com (X.D.); 20221012@zcmu.edu.cn (Y.S.); 2 Key Laboratory for Collaborative Innovation and Sustainable Utilization of Dao-di Herbs, Songyang Institute, Zhejiang Chinese Medical University, Lishui 323400, China

**Keywords:** *Dendrobium officinale*, GATA, expression analysis, flavonoid, stress

## Abstract

*Dendrobium officinale,* which was rich in bioactive compounds such as polysaccharides, alkaloids, amino acids, and flavonoids, had significant medicinal value and ability to resist stresses. Studies had demonstrated that *GATA* genes were one of the crucial regulators in controlling plant development and growth and stress response. Genome-wide identification and characterization of the 18 *DoGATA* genes were displayed. According to phylogenetic relationships, the *DoGATA* family genes were divided into 4 groups and the conserved motifs of *DoGATA1*–*DoGATA18* within the same group were similar. All *DoGATA* genes were localized in the nucleus and randomly mapped on 10 chromosomes. The GATA genes in *D. officinale* experienced one pair of tandem duplication and 4 pairs of segment duplications to expand the family genes. Additionally, we found that the 2000 bp upstream promoter region of the *DoGATA* genes harbored 23 types of *cis*-acting elements that were categorized into plant growth and development, phytohormone responsiveness, and stress responsiveness. *DoGATA1*–*DoGATA18* were diversely expressed across different tissues (root, leaf, stem, flower), exposed to salt stress, and following MeJA treatment. Co-expression analysis between *DoGATA* and enzyme-encoding genes involved in the biosynthesis of flavone showed that *DoCHI* (LOC110104562) and *DoGTMT* (LOC110098370) may be potential downstream targets of *DoGATA16* to regulate flavonoid biosynthesis to adapt to salt stress. Furthermore, we confirmed that *DoGATA16* may act as a key member to resist stress. The collective findings of this study shed light on the function of *GATA* genes and molecular breeding of *D. officinale*.

## 1. Introduction

Plants exposed to diversity abiotic stresses (e.g., light, drought, extreme temperature, salinity, etc.) or biotic stress (e.g., insect attacks, virus infiltrates, weed hazards, etc.), often experience oxidative stress, impairing growth or causing injury and death [1]. Extensive studies have investigated the molecular mechanisms that enable plants to respond to stress [2]. Flavonoids, polyphenolic secondary metabolites, served as crucial stress mitigators to alleviate these stresses by modulating redox balance, enhancing signaling, and regulating stress-responsive transcription factors [3]. For example, *Camellia sinensis* significantly enhanced the drought tolerance by accelerating the biosynthesis of flavonoids in plants [4]. *Ginkgo biloba MYB11* bind and activate flavonoid biosynthesis-related genes [flavanone-3′-hydroxylase (*F3*’*H*) and flavanol synthase (*FLS*)] to improve their salt tolerance [5].

Moreover, chemical elicitors further amplify this defense mechanism. For example, as a core signaling molecule in the metabolic pathway, methyl jasmonate (MeJA, one of the best derivatives of JA) reduced cadmium toxicity by enhancing flavonoid metabolism in *Cajanus cajan* [6]. Additionally, MeJA alleviated UV-B-induced stress signaling by modulating endogenous hormones in plants, thereby enhancing accumulation of the anthocyanins [7]. After ABA treatment, pigeon pea up-regulated the expression of the key enzyme genes in the pathway of flavonoids and improved the accumulation of flavonoids remarkably [8].

GATA was a class of transcription factors (TFs) that contained one or two conserved type IV zinc finger domains (C-X_2_-C-X_17–20_-C-X_2_-C), which specifically recognized and bind the WGATA/R motif (W = T/A; R = G/A) to regulate downstream gene transcription [9]. Nowadays, GATA proteins have been comprehensively identified and analyzed in many plants, which are divided into four groups based on their evolutionary relationships and genes structures [10]. In *Zea mays*, among the 41 *GATA* genes, *ZmGATA37* was differentially expressed under excessive temperatures and salt stress, suggesting that *ZmGATA37* may function as a key regulator in stress resistance [11]. *Phyllostachys edulis GATA26* significantly regulated the growth of roots and stems by increasing the expression of GA3 oxidase and decreasing the expression of GA2 oxidase [12]. In *Ipomoea batatas*, *IbGATA24* interacted with *IbCOP9-5a* to enhance drought and salt tolerance through regulating the ABA and JA signaling pathway-related genes expression [13].

*Dendrobium officinale* Kimura et Migo, an orchidaceae medicinal herb widely distributed in southern China, produced valuable secondary metabolites (polysaccharides, flavonoids, alkaloids) with various pharmacological activities [14,15,16]. This epiphytic plant thrived in diverse environments (1000–3000 m altitude), highlighting its stress adaptation capacity. However, despite *GATA* genes’ known role in plant developmental processes and stress-response mechanisms, this gene family remains uncharacterized in *D. officinale*.

In the present study, we organized a systematic investigation of the *GATA* genes family in *D. officinale* based on its high-quality genome. The protein physicochemical properties, gene structure, evolutionary relationships, chromosome localization, collinearity, *cis*-regulating elements, and relative expression of different organs were comprehensively analyzed. Furthermore, the expression pattern of GATA family members of *D. officinale* was determined under salt stress and MeJA treatment. Subsequently, we constructed a co-expression between *DoGATA* genes, and the enzyme-encoding genes involved in flavonoid biosynthesis. Generally, this study established *DoGATA* gens as critical regulators relating to JA signaling and salt stress response in *D. officinale*, providing molecular targets for enhancing stress tolerance in this valuable medicinal orchid.

## 2. Results

### 2.1. Genome-Wide Identification and Physicochemical Properties Analysis of DoGATA Proteins

**Figure 1:**
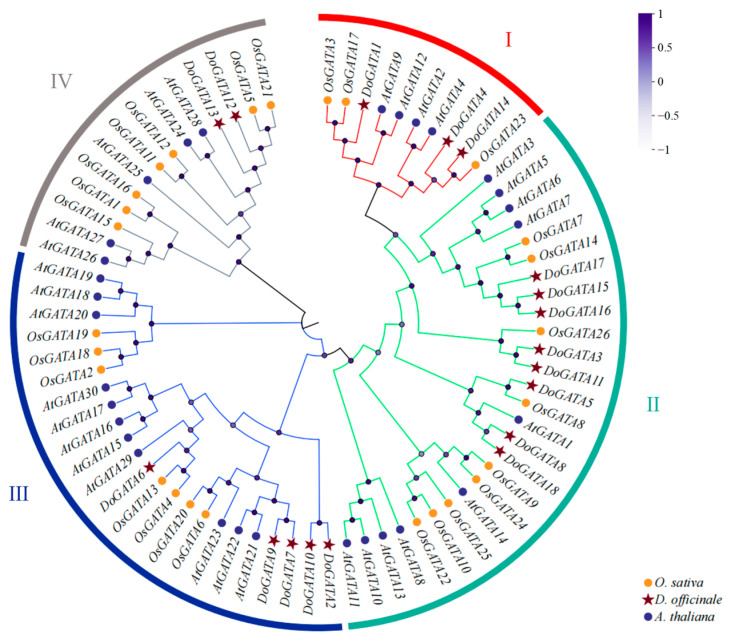
Phylogenetic relationship between the 18 *DoGATA*, 30 *OsGATA*, and 26 *AtGATA* genes. The phylogenetic tree was constructed using the neighbor-joining method with 1000 bootstrap values. The varying degrees of purple dots on the branches represent different bootstraps. The red, green, blue, and grey rings indicate Group I, II, III, and IV, respectively.

Through HMMER analysis, and conserved domains identification, 18 *DoGATA* genes were finally screened from the genome of *D. officinale*. Based on their position across the 10 chromosomes, *DoGATA* were identified as *DoGATA1–DoGATA18* (Figure 1). ExPASy analysis showed that the number of GATA proteins amino acids was between 140 and 756 aa, with an average amino acid number of 305 aa (Table 1). The molecular weight varied from 15.31 (DoGATA5) to 85.03 kDa (DoGATA7). The isoelectric points (pI) of the proteins ranged from 5.40 to 9.97. Among them, the pI of DoGATA1, DoGATA4, DoGATA5, DoGATA13, and DoGATA14 were less than 7, suggesting that they were acidic protein. All DoGATA proteins with an instability index > 40 were predicted to be unstable, whereas the aliphatic index ranged from 50.76 (DoGATA3) to 77.08 (DoGATA9). The GRAVY of individual DoGATA proteins was consistently negative, predicting their intrinsic hydrophilic characteristics. Subcellular localization results implied that all DoGATA were localized on the nucleus, indicating that these proteins might function as TFs.

### 2.2. Phylogenetic Analysis of DOGATA Proteins

To explore the classification and phylogenetic relationship of GATA proteins in different plants, 18 *D. officinale* GATA proteins, 26 *O. sativa* GATA proteins, and 30 *A. thaliana* GATA proteins were recruited to establish an evolutionary tree (Figure 1). According to their phylogenetic relationships, these 74 DoGATA proteins were completely separated into four groups: group I, group II, group III, and group IV made up of 10, 27, 23, and 14 proteins, respectively.

Notably, GATA proteins in either monocots or dicots are present in each group. Meanwhile, the DoGATA proteins showed an uneven distribution pattern among these four groups. In *D. officinale*, 8/18 of the GATA proteins (DoGATA3, DoGATA5, DoGATA8, DoGATA11, DoGATA15, DoGATA16, DoGATA17, and DoGATA18) were clustered in group II. Group III had 5 members (DoGATA2, DoGATA6, DoGATA7, DoGATA9, and DoGATA10), followed by groups I (DoGATA1, DoGATA4, and DoGATA14) and group IV (DoGATA12 and DoGATA13).

Furthermore, phylogenetic analysis revealed distinct evolutionary patterns between monocot and dicot GATA proteins. Monocot GATA proteins consistently clustered together on the same branch, suggesting that GATA proteins in monocots and dicots exhibit a degree of conservation during evolution.

### 2.3. Multiple Sequence Alignment, Motifs and Structure Analysis of DOGATA Proteins and Genes

**Figure 2:**
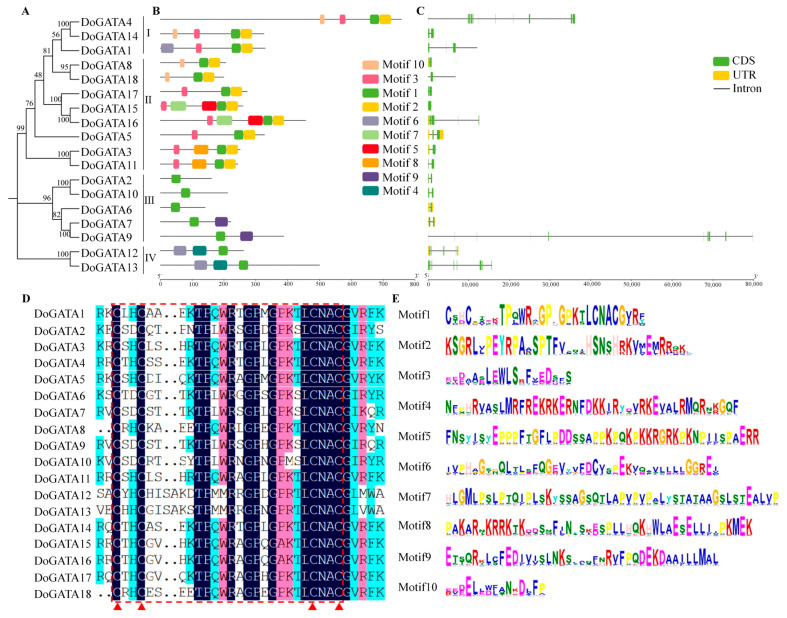
(**A**) Phylogenetic tree of the *DoGATA* gene family. (**B**) Conserved motifs of *DoGATA* genes. The *X*-axis represents the protein molecular weight (kDa) of each gene. (**C**) The structure of the *DoGATA* genes. The *X*-axis represents the genes’ length (bp). (**D**) Multiple sequence alignment. The red lines and triangle highlight the conserved GATA domain (C-X_2_-C-X_17–20_-C-X_2_-C). (**E**) Ten conserved motifs of *DoGATA* genes.

To elucidate the features of DoGATA proteins, conserved motifs (*n* = 10) were performed. Notably, multiple sequence alignment revealed that motif 1 represented a unique motif in all DoGATA proteins, containing the characteristic conserved domain. GATA proteins in the same group almost significantly contained correlated motifs (Figure 2A,B). Motif 1 and motif 2 clustered appeared in groups I and II, while motif 3 and motif 10 only partially existed in group I and group II, which indicating that these genes may duplicate along the way to play the same important functions. Motifs 5/8 were found in group II, while motif 9 was found in group III. Except for motifs 1/4/6, group IV no longer contained other conserved motifs.

Gene structure analysis (Figure 2C) revealed a variation in coding sequence (CDS) organization among DoGATA family members. Notably, 6 of 18 genes *(DoGATA3*, *DoGATA5*, *DoGATA6*, *DoGATA7*, *DoGATA8*, and *DoGATA12*) contained untranslated regions (UTRs) N-terminus and the C-terminus, while the remaining genes showed distinct CDS architectures.

Multiple sequence alignment performed by DNAMAN v9.0 were displayed to explore the characterization of *D. officinale* GATA proteins (Figure 2D). The result showed all DoGATA proteins consist of 50–53 highly conserved domains (C-X_2_-C-X_17–20_-C-X_2_-C) in the N- terminal, suggesting that GATA proteins in *D. officinale* were conserved during the evolution and may perform similar functions.

To characterize DoGATA protein features, we identified 10 conserved motifs through comprehensive analysis. Notably, multiple sequence alignment revealed that motif 1 represents a universal signature present in all DoGATA proteins, containing the characteristic conserved domain (Figure 2E).

Moreover, the distribution of introns was also varied. The intron regions of *DoGATA15* and *DoGATA17* are relatively short, whereas *DoGATA9* possesses the longest intron region. Overall, 18 *GATA* genes in *D. officinale* performed varied organization, which also corresponded to their multiple functions.

### 2.4. Chromosome Localization and Collinearity Analysis of DoGATA Genes

**Figure 3:**
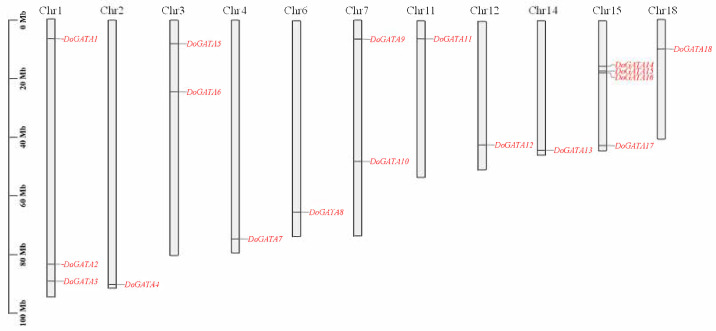
Chromosomal localization of *GATA* genes in *D. officinale*. The positions of *DoGATA* gene members were marked on Chr 1, 2, 3, 4, 6, 7, 11, 12, and 14. The left axis indicates chromosome length (Mb).

According to the annotated files of *D. officinale* genome, *DoGATA1*–*DoGATA18* chromosome localization was performed. As shown in Figure 3, *D. officinale* possessed 19 chromosomes, and the length of the chromosomes were varied. Chr1 was the longest chromosome, while Chr19 was the shortest. *DoGATAs* were unevenly distributed across Chr 1, 2, 3, 4, 6, 7, 11, 12, 14, 15, and 18. Chr 15 mapped 4 members (*DoGATA14–DoGATA17*) which were the chromosome that contain the largest number of genes. Chr 1 had 3 members, Chr 3 and 6 had 2 members, and Chr 2, 4, 7, 11, 14, and 18 had only 1 member.

**Figure 4:**
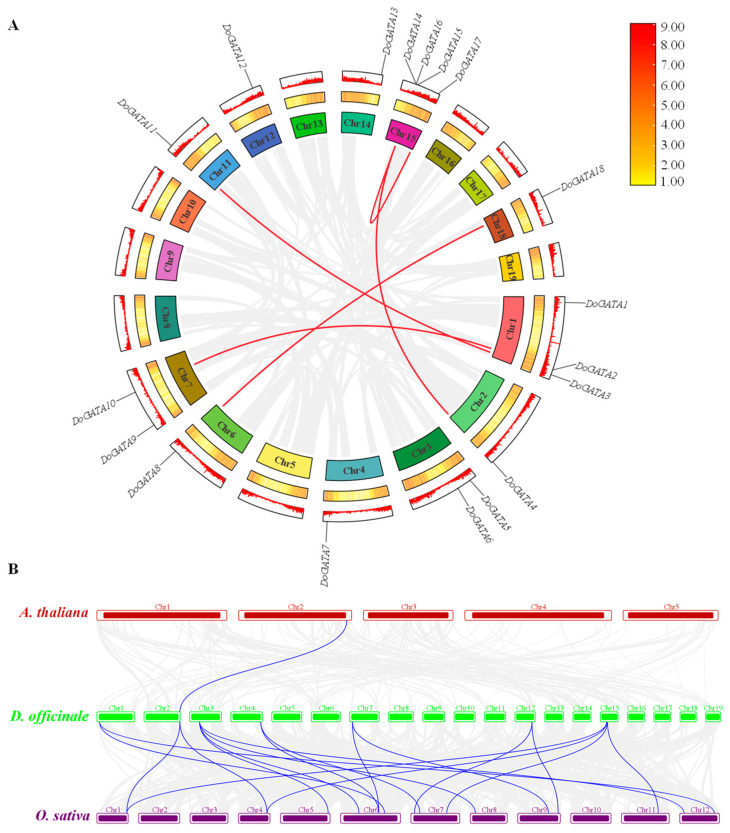
Collinearity analysis of *DoGATA* genes. (**A**) Intergroup collinearity analysis of *DoGATA* genes. Gene density was displayed in both linear and heatmap formats. (**B**) Collinearity of *DoGATAs*, *A. thaliana*, and *O. sativa*.

Additionally, we visualized the gene density of *D. officinale* across both heatmap and linear pattern (Figure 4A), and the results demonstrated that all *GATA* genes are localized in regions with relatively high gene density on the chromosomes. Intra-species collinearity implied that there were 5 pairs of genes replication events (*DoGATA2*–*DoGATA10*, *DoGATA3*–*DoGATA11*, *DoGATA4*–*DoGATA15*, *DoGATA16*–*DoGATA17*, and *DoGATA8*–*DoGATA18*) within the entire *DoGATA* genes family. In addition, of these gene pairs, *DoGATA8*–*DoGATA18* and *DoGATA16*–*DoGATA17* belong to group II, *DoGATA3*–*DoGATA11* and *DoGATA2*–*DoGATA10* belong to group III, whereas *DoGATA4*–*DoGATA15* did not belong to the same group.

Simultaneously, the *GATA* genes in *D. officinale* had inter-species collinearity with model plants *A. thaliana* (*n* = 1) and *O. sativa* (*n* = 18), respectively (Figure 4B), which corresponded to the fact that *D. officinale* and *O. sativa* are both monocots plant. These findings provided valuable insights into the conservation and divergence of GATA gene family organization across major plant lineages.

### 2.5. Cis-Acting Elements Analysis of DoGATA Genes

**Figure 5:**
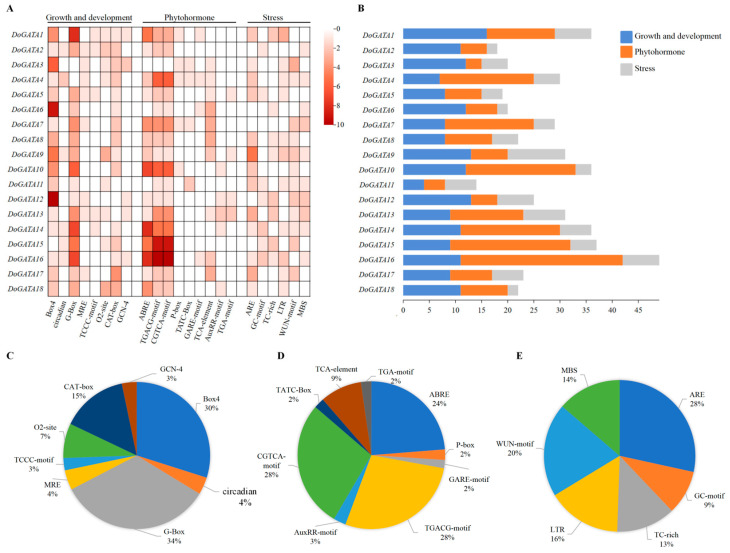
*Cis*-acting elements (CAEs) analysis of *GATA* genes in *D. officinale.* (**A**) Heatmap of CAEs in the promoter sequences of *DoGATA* genes responding to 3 categories. The number of each CAE was visualized as a heatmap. (**B**) Number of CAEs in each *DoGATA* gene. (**C**) Histogram of different CAEs in growth and development. (**D**) Histogram of different CAEs in phytohormone. (**E**) Histogram of different CAEs in stress.

*Cis*-acting elements (CAEs) with specific DNA sequences play an important role in the regulation of gene expression. They modulated transcriptional activity by interacting with TFs. In total, 23 types of CAEs were predicted by the PlantCare database and categorized into three categories: Plant growth and development, Phytohormone, and Stress (Figure 5A). The promoters’ numbers of each *DoGATA* gene were different, while *DoGATA16* contained the largest number (*n* = 49) and *DoGATA11* contained the least number (*n* = 14) of CAEs. CAEs relating to phytohormone responsiveness occupied the largest proportion (*n* = 219) (Figure 5B). Among them, MeJA responsive elements (TGACG-motif and CGTCA-motif) and ABA responsive element (ABRE) were present in almost all promoter of *DoGATA* genes (Figure 5A).

Apart from these, 8 CAEs related to plant growth and development were identified (Figure 5C). Among them, light related elements (G-box, Box-4, TCCC-motif, circadian, and MRE) were widely distributed among the *DoGATA* genes, which suggested that *DoGATA* may play an important regulatory role in light regulation. CAT-box (meristem expression related), GCN-4 (endosperm expression related), and O2-site (zein metabolism regulation related) were also displayed in some *DoGATA* genes, suggesting that *DoGATA* genes potentially regulated the growth and development of *D. officinale*. In addition, the TCA-element which responded to salicylic acid (SA) also presented in large quantities in the promoters of *DoGATA* genes. These results claim that the *DoGATA* genes may be regulated by phytohormone to affect the accumulation of active metabolites (Figure 5D). Moreover, *DoGATA* family members also had massive MBS, LTR, and ARE CAEs that responded to stress responsiveness (Figure 5E).

### 2.6. Structural Characterization of DoGATA Proteins at Secondary and Tertiary Levels

The SOPMA and SWISS-MODEL were used to confirm the protein secondary and tertiary structure of DoGATAs. The results showed that the secondary structure of GATA proteins in *D. officinale* consists of α-helix, β-turn, random coil, and extended strand. Among these different protein structures, random coil account for the largest proportion (36.25–74.26%) in all GATA proteins followed by α-helix (13.90–34.38%), extended strand (4.98–21.33%), and β-turn (3.08–10.00%) (Table 2).

**Figure 6:**
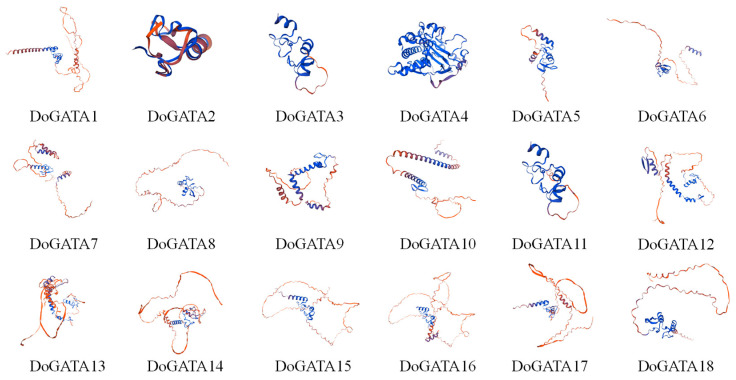
Tertiary structure analysis of DoGATA proteins. Red indicated α-helices, yellow represented β-sheets, and blue denoted random coil.

Additionally, the tertiary structure of DoGATA1–DoGATA18 revealed that all proteins contained α-helix, β-turn, random coil, and extended strand which was consistent with the secondary structure (Figure 6). While tertiary structures showed some variation among members, each protein maintained the characteristic GATA conserved domain (Figure 2D).

### 2.7. Expression Patterns in Different Tissues of DoGATA Genes

**Figure 7:**
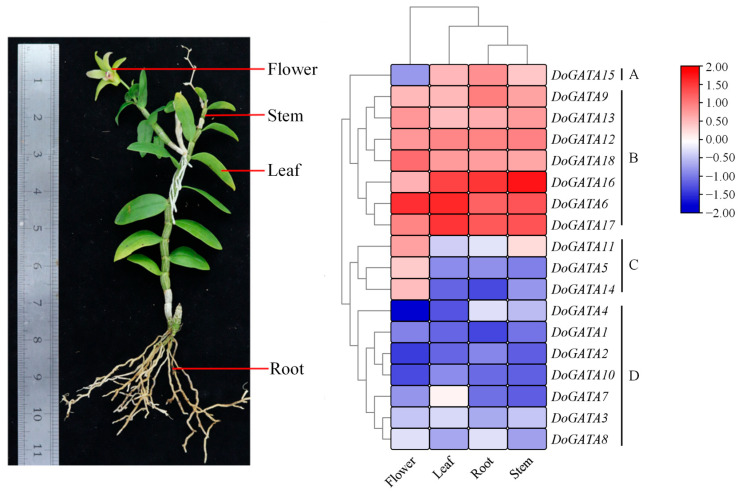
Expression pattern analysis of *DoGATA* genes in different tissues (flower, leaf, root, and stem), as determined by qRT-PCR.

To investigate their functional roles, we analyzed the expression patterns of *D. officinale* GATA genes across four tissues (root, stem, leaf, and flower) using qRT-PCR (Figure 7). Cluster analysis based on the heatmap revealed 4 distinct expression patterns (Type A, B, C, and D) among *DoGATA1*–*DoGATA18* members, suggesting functional diversification within *DoGATA* genes.

The major genes (*DoGATA6/9/12*/*13*/*16*/*17*/*18*) were clustered in Type B and they were highly expressed in all tissues, suggesting their potential function in plants growth and development. Specially, *DoGATA6/16*/*17* showed predominant expression in leaf, root, and stem, while *DoGATA6* additionally exhibited in flower. In contrast, the genes in Type C (*DoGATA5/11/14*) had the highest expression in flower but were nearly undetectable in other tissues. Meanwhile, *DoGATA15* in Type A was expressed in all tissues except the flower. The remaining 8 genes in Type D had low expression across all tissues, forming a non-specific expression group.

### 2.8. Expression Patterns Under Salt and MeJA Treatment of DoGATA Genes

Salinity, as a main environmental factor, created salt stress when accumulation levels exceeded the tolerance of the plants. Salt stress had various effects on plants. High concentrations of salts disrupted the osmotic balance of plant cells, leading to accelerated water loss and stomatal closure, which consequently impaired photosynthetic efficiency. In addition, salt stress disrupted the ion balance within plants, resulting in toxic accumulation of metal ions. In addition to these, salt stress also interfered with the absorption of essential nutrients such as nitrogen and phosphorus, limiting growth and metabolism [17]. Generally, roots and leaves were the main tissues to adapt to salt stress by sequestering Na^+^ or Cl^−^ and accumulating metabolites to regulate osmotic pressure. Moreover, plants can also react against salt stress by activating signal transduction, modulating transcriptional regulation, and inducing stress-responsive gene expression [18].

**Figure 8:**
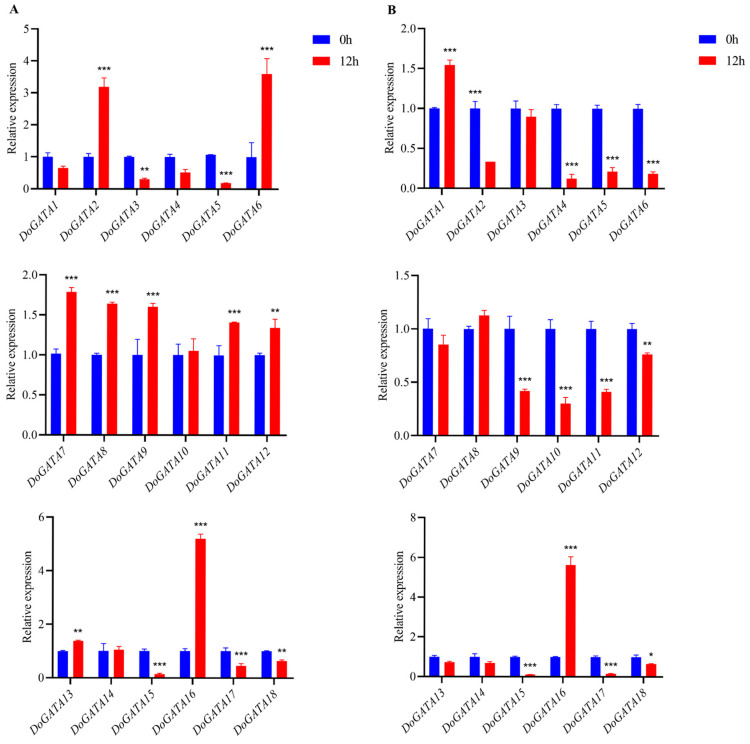
Expression levels of *DoGATA* genes under salt-treated in leaf (**A**) and root (**B**) for 12 h. Mean values and standard deviations (SDs) indicated by error bars. Significant differences: * (*p* < 0.05), ** (*p* < 0.01), and *** (*p* < 0.001).

The *DoGATA* genes have been demonstrated to play notable regulatory roles in multiple biological processes, including plant growth and development, and stress adaptation. We used qRT-PCR to examine the relative expression levels of *DoGATA* genes in *D. officinale* salt-treated leaves and roots under 12 h 250 mM. In leaves (Figure 8A), the relative expression levels of 1/2 number of *DoGATA* genes (*DoGATA2*, *DoGATA6*, *DoGATA7*, *DoGATA8*, *DoGATA9*, *DoGATA11*, *DoGATA12*, *DoGATA13*, *DoGATA16*) were significantly increased. Notably, *DoGATA16* showed significant up-regulation with 5.19-fold. *DoGATA1*, *DoGATA3*, *DoGATA4*, *DoGATA5*, *DoGATA10*, *DoGATA14, DoGATA15*, *DoGATA17*, and *DoGATA18* showed different degrees of down-regulated expression levels after salt-treated in leaves. However, in roots, only *DoGATA1*, *DoGATA8*, and *DoGATA16* up-regulated, ranging from 1.32 to 5.63 (Figure 8B).

Conversely, the remaining 15 genes exhibited decreased expression levels compared with the untreated group. Overall, expression levels of *DoGATA1*–*DoGATA18* were broadly split into three groups: up-regulated expression in both leaves and roots (*DoGATA8* and *DoGATA16*), down-regulated expression in both leaves and roots (*DoGATA3*/*4*/*5*/*15*/*17*/*18*), and inconsistent expression pattern in leaves and roots (*DoGATA1*/*2*/*6*/*7*/*9*/*10*/*11*/*12*/*13*/*14*/*16*).

As a natural plant hormone, MeJA served as a key signaling molecule with significant regulatory roles in various physiological processes, including seed germination, growth and development, leaf senescence, and metabolite accumulation in plants. Moreover, when plants are subjected to various stresses, MeJA can protect plants through different association regulation pathways within an appropriate timeframe and enabling adaptation to the environment.

**Figure 9:**
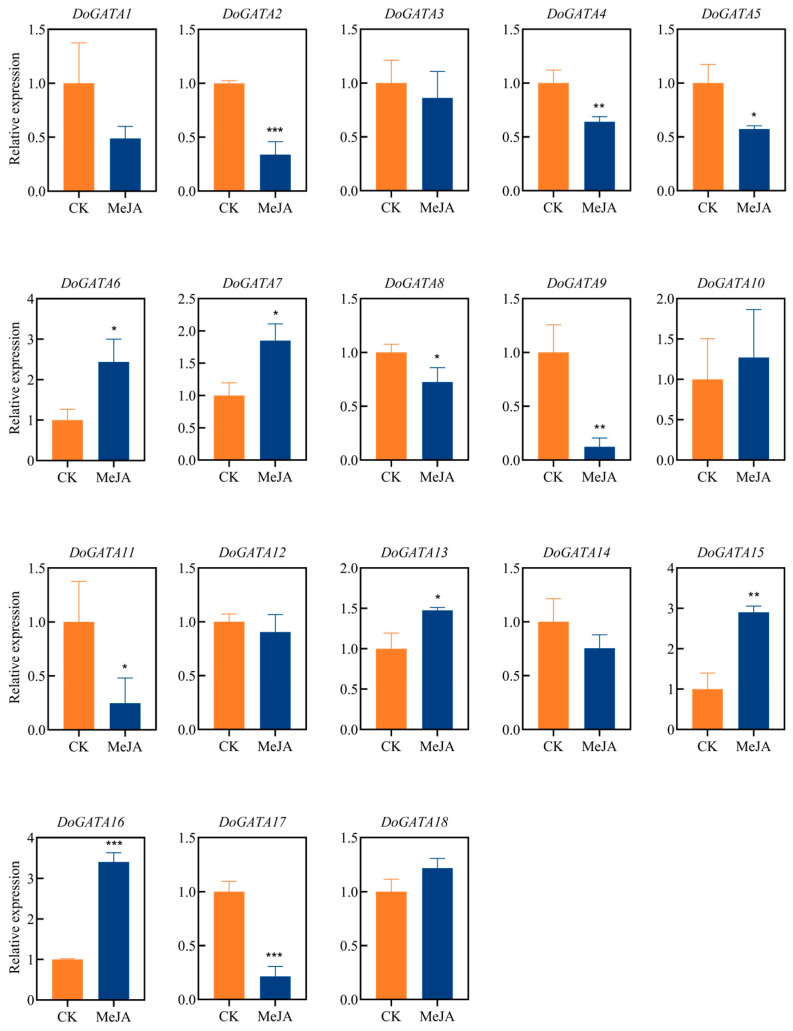
Expression levels of *DoGATA* genes under MeJA-treatment in leaf for 12 h. Mean values and standard deviations (SDs) indicated by error bars. Significant differences: * (*p* < 0.05), ** (*p* < 0.01), and *** (*p* < 0.001).

Since abundant MeJA-related motifs existed in the upstream 2000 bp of *DoGATA*1*–DoGATA18,* we examined their dynamic expression patterns in response to MeJA treatment using qRT-PCR analysis (Figure 9). The expression level of *DoGATA* genes showed relative variance. The expressions levels of seven *DoGATA* genes (*DoGATA6*, *DoGATA7*, *DoGATA10*, *DoGATA13*, *DoGATA15*, *DoGATA16*, *DoGATA18*) were up-regulated. In particular, *DoGATA16* showed the most obvious up-regulation by more than 3-fold. However, the remaining *DoGATA* genes (*DoGATA1*, *DoGATA2*, *DoGATA3*, *DoGATA4*, *DoGATA5*, *DoGATA8*, *DoGATA9*, *DoGATA11*, *DoGATA12*, *DoGATA14*, and *DoGATA17*) decreased 0.12–0.85-fold under MeJA treatment.

### 2.9. Correlation Analysis Between DoGATA Genes and Enzyme-Encoding Genes Involved in the Biosynthesis of Flavonoid

**Figure 10:**
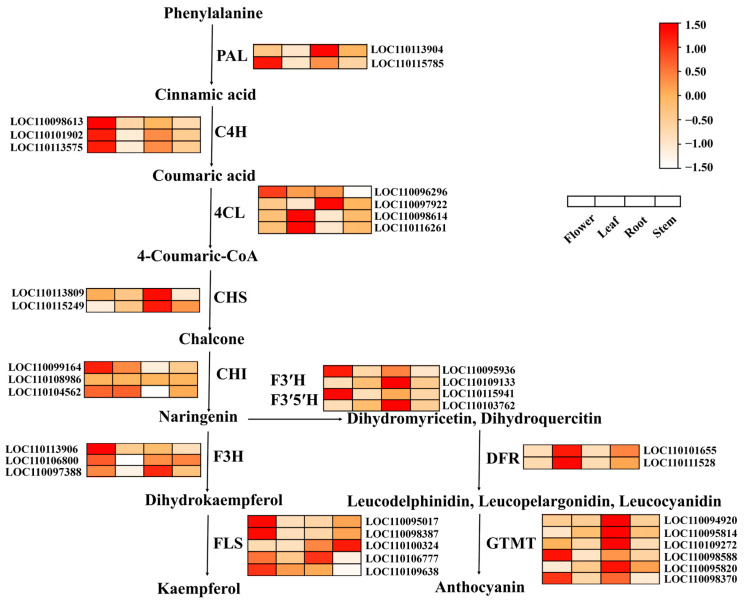
Expression profiles of enzyme-encoding genes involved in flavonoid biosynthesis in the different tissues (flower, leaf, root, and stem). *DoPAL* (LOC110113904, LOC110115785): phenylalanine ammonia-lyase. *DoC4H* (LOC110098613, LOC110101902, LOC110113575): cinnamate 4-hydroxylase. *Do4CL* (LOC110096296, LOC110097922, LOC110098614, LOC110116261): 4-coumarate coenzyme A ligase. *DoCHS* (LOC110113809, LOC110115249): chalcone synthase. *DoCHI* (LOC110099164, LOC110108986, LOC110104562): chalcone isomerase. *DoF3*′*H* (LOC110095936, LOC110109133, LOC110115941): flavanone-3′-hydroxylase. *DoF3*’*5’H* (LOC110103762): flavanone-3′,5′-hydroxylase. *DoF3H* (LOC110113906, LOC110106800, LOC110097388): flavanone-3-hydroxylase. *DoFLS* (LOC110095017, LOC110098387, LOC110100324, LOC110106777, LOC110109638): flavone synthase. *DoDFR* (LOC110101655, LOC110111528): dihydroflavonol 4-reductase. *DoGTMT* (LOC110094920, LOC110095814, LOC110109272, LOC110098588, LOC110095820, LOC110098370): anthocyanidin 3-O-glucosyltransferase.

In *D. officinale*, flavonoid biosynthesis primarily occurred across the phenylpropanoid pathway, a key metabolic route that generated diverse flavonoid compounds (Figure 10).

The flavonoid biosynthesis pathway in *D. officinale* began with phenylalanine deamination catalyzed by phenylalanine ammonia-lyase (PAL), yielding cinnamic acid. Subsequently, cinnamic acid produced coumaric acid mediated by cinnamate-4-hydroxylase (*C4H*). The 4-coumarate-CoA ligase (*4CL*) converted coumaric acid to 4-Coumaric-CoA which was catalyzed by chalcone synthase (*CHS*) to generate chalcone. Chalcone was then catalyzed by chalcone isomerase (*CHI*) to form naringenin. On the one hand, with the assistance of flavanone 3-hydroxylase (*F3H*) and flavanol synthase (*FLS*), naringenin was ultimately converted to kaempferol. On the other hand, flavonoid 3′-hydroxylase (*F3*’*H*), flavonoid 3′,5′-hydroxylases (*F3*’*5*’*H*), dihydroflavonol 4-reductase (*DFR*), and glycosyltransferase (*GTMT*) also generated naringenin to anthocyanin.

We identified enzyme-encoding genes involved in the biosynthesis of flavonoid and detected the expression pattern of these genes in different tissues (flower, root, leaf, and stem). *DoPAL* (LOC110113904, LOC110115785), *DoF3H* (LOC110113906, LOC110106800, LOC110097388), *DoC4H* (LOC110098613, LOC110101902, LOC110113575), *DoF3*’*H* (LOC110095936, LOC110109133, LOC110115941), *DoF3*′*5*′*H* (LOC110103762), and *DoGTMT* (LOC110094920, LOC110095814, LOC11010927, LOC11009858, LOC110095820, LOC110098370) were highly expressive in flower and root. Furthermore, *Do4CL* (LOC110096296, LOC110097922, LOC110098614, LOC110116261) and *DoCHI* (LOC110099164, LOC110108986, LOC110104562) displayed high expression in flower, leaf, and root. *DoDFR* (LOC110101655, LOC110111528) was only highly expressed in leaf and *DoFLS* (LOC110095017, LOC110098387, LOC110100324, LOC110106777, LOC110109638) was highly expressed in each tissue apart from leaf.

**Figure 11:**
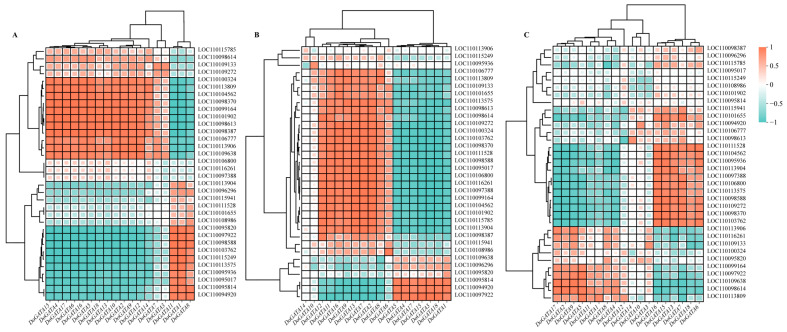
Correlation analysis with enzyme-encoding genes involved in flavonoid biosynthesis and *DoGATA* genes under salt-treated in leaf (**A**), root (**B**), and MeJA-treated (**C**).

To investigate how *DoGATA* genes resist stress, correlation analysis between *DoGATA* genes and enzyme-encoding genes involved in the biosynthesis of flavonoid by salt and MeJA treatment was performed (Figure 11). In salt-treated leaf (Figure 11A), *DoGATA2*/*6*/*7*/*8*/*9*/*11*/*12*/*13*/*16* had a significant co-expression relationship with most of the enzyme-encoding genes and so did *DoGATA2*/*3*/*4*/*5*/*6*/*7*/*9*/*10*/*12*/*13*/*14*/*15*/*16*/*17*/*18* in salt-treated root (Figure 11B). After MeJA treatment, *DoGATA* genes exhibited various degrees of correlation with flavonoid biosynthesis genes (Figure 11C). These collective results demonstrated the functional diversity of GATA genes in regulating the flavonoid biosynthesis pathway.

**Figure 12:**
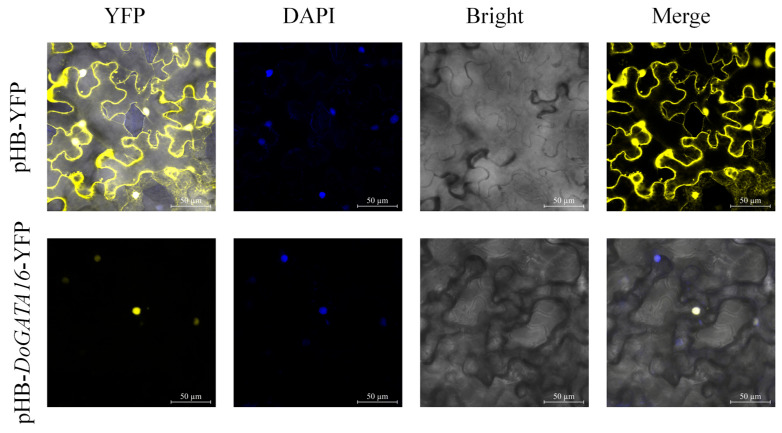
Subcellular localization of *DoGATA16*. The pHB-YFP acted as a control.

Combined with the correlation analysis between *DoGATA16* and enzyme-encoding genes involved in the biosynthesis of flavone, we found *DoGATA16* may potentially bind to the possible downstream targets (LOC110104562 and LOC110098370) to influence the accumulation of flavonoids in *D. officinale* and enhance its ability to tolerate stress. Based on this, we performed subcellular localization of *DoGATA16*. The results demonstrated that YFP is a fluorescent protein localized to both the nucleus and the plasma membrane. Subcellular localization analysis revealed that pHB-*DoGATA16*-YFP was localized in the nucleus which was consistent with the predicted results (Figure 12).

## 3. Discussion

Transcription factors are a class of DNA-binding proteins that recognize specific *cis*-acting elements to regulate target gene expression. Among these, the GATA proteins contains a motif which was widely identified in eukaryotes. In animals, GATA proteins typically contain two zinc finger domains [19], whereas in plants, the majority of GATA proteins possess a single highly conserved type IV zinc finger. Currently, the GATA gene family is not only regulating the growth and development of plants but also plays a crucial regulatory role in responding to adverse stress such as salinity, drought, and high temperatures [20]. Extensive research on GATA genes has been conducted in model plants, including *A. thaliana* (*n* = 30) [21], *O. sativa* (*n* = 27) [21], and *S. lycopersicum* (*n* = 30) [22], with significant progress made in understanding their functional and regulatory mechanisms. Additionally, GATA family genes have been systematically identified in various other species. including *Cucumis melo* (*n* = 24) [23], *Solanum tuberosum* (*n* = 36) [24], *Dimocarpus longan* (*n* = 22) [9,25], *Phaseolus vulgaris* (*n* = 31) [26], and *Platycodon grandiflorum* (*n* = 22) [27].

Here, we identified and excavated 18 *DoGATA* family genes from high-quality genome of *D. officinale*, named *DoGATA1*–*DoGATA18*. The amino acid number of *DoGATA* genes ranged from 140 to 756 aa (Table 1) exhibited comparable size variation to orchid GATA proteins [28], but exhibited more variation than GATA genes in other species such as *P. edulis* [12], *P. vulgaris* [26], and *D. longan* [9]. Apart from this, *DoGATA1–DoGATA18* proteins also displayed significant differences in MW, II, and AI (Table 1). Notably, all DoGATAs were hydrophilic proteins, a feature shared with *Vitis vinifera* GATA proteins [29], suggesting their potential functional significance in environmental stress responses.

All DoGATA proteins contained conserved motif 1 (Figure 2D), consisting of GATA domains, which were confirmed in *Eucalyptus urophylla* [30], *Moso Bamboo* [12]. To explore the evolutionary relationships of the GATA proteins, 74 proteins including 30 *A. thaliana* GATA proteins, 26 *O. sativa* GATA proteins, and 18 *D. officinale* GATA proteins were considered in a phylogenetic tree (Figure 1). According to the evolutionary relationships, the *D. officinale* GATA proteins were categorized into four groups: A, B, C, and D. Moreover, the *DoGATA* genes predominantly clustered with *OsGATA* genes, whereas the *AtGATAs* always formed a separate cluster, suggesting that GATA genes of *D. officinale* and *O. sativa* may perform similar functions [31].

Among the 19 chromosomes of *D. officinale*, *DoGATA* genes were unevenly distributed on 10 chromosomes (Chr 1, Chr 2, Chr 3, Chr 4, Chr 6, Chr 7, Chr 11, Chr 14, Chr 15, Chr 18), and Chr 15 carried the most *DoGATA* genes (*DoGATA*14, *DoGATA*15, *DoGATA*16, *DoGATA*17) (Figure 3). Moreover, in the process of evolution, 1 pair of genes (*DoGATA16*–*DoGATA17*) obtained tandem duplication to promote the expansion of the gene family and 5 pairs of genes (*DoGATA2*–*DoGATA10*, *DoGATA3*–*DoGATA11*, *DoGATA4*–*DoGATA15*, and *DoGATA8*–*DoGATA18*) segmentally duplicated to promote more diverse functions (Figure 4A). Furthermore, the collinearity between *D. officinale* and *A. thaliana* or *O. sativa* was 1 pair and 18 pairs, respectively, which is consistent with the evolutionary pattern of dicotyledonous plants (Figure 4B). Generally, the genetic diversification of GATA family genes, including genes duplication and location variation, drove plants to optimize their specific survival environment [32].

The tissue-specific expression patterns of *DoGATA* genes reflected their functional diversification. All 18 *DoGATA* genes performed various expression patterns in four tissues (root, leaf, flower, stem) (Figure 7). Previous studies have identified that *AtGATA25* and *AtGNC* (A type of GATA TF) regulated flowering time, flowering pigmentation, and fragrance biosynthesis [31,33]. In this study, we observed genes in Type A/B/C excluding *DoGATA15* exhibiting high expression in flower, which strongly suggested that DoGATA genes may have the same function. Moreover, the results revealed that DoGATA6/16/17 exhibited constitutively high expression across all tissues, resembling the expression patterns of functionally characterized *ZmGATA* genes involved in growth and development [11]. Specially, *DoGATA16* exhibited the highest stem-specific expression, indicating its potential regulatory role in accumulating the bioactive secondary metabolites.

Recent studies have revealed that the SlGATA17-SIHY5 protein complex enhanced salt tolerance in *Solanum lycopersicum* [34]. *StGATA12* regulated the levels of H_2_O_2_, malondialdehyde (MDA) to reinforce the tolerance of salt stress and osmosis-induced damage [35]. Consistent with these findings, we exposed *D. officinale* leaf and root to salt stress and found *DoGATA* genes displayed various expression patterns (Figure 8A,B), which may be associated with the presence of stress-related *cis*-acting elements in these genes. Among these, the most notable observation was that the expression levels of *DoGATA16* increased by more than 4- and 5-fold under salt stress. The results indicated that the majority of *DoGATA* genes containing TGACG-motif and CGTCA-motif appeared to be responsive to MeJA regulation (Figure 5). Following 12 h of MeJA treatment, 39% of *DoGATA* genes exhibited diversity up-regulation (Figure 9). Notably, *DoGATA16* showed the most significant up-regulation with a 3.42-fold increase in expression level which had the most MeJA-related *cis*-elements. In summary, *DoGATA16* exhibited significant up-regulation in expression under both salt stress and MeJA induction, suggesting its potentially critical role for defending salt stress and playing an essential role in the MeJA-associated regulatory network.

Flavonoids up-regulated the activity of endogenous antioxidant enzymes, directly reducing ROS and binding some metal ions [36,37] as a defense to a series of stresses. MeJA was a vital signaling molecule in response to drought stress, which induced stomatal closure and activated the antioxidant system [38] to significantly enhance plant adaptation to stress. To investigate how *DoGATA* genes resist stress, we conducted a correlation analysis (Figure 11). *DoGATA16* showed significant positive correlations with multiple flavonoid biosynthesis related genes and it can be inferred that it may play an essential regulatory role in the flavonoid biosynthesis pathway, particularly under salt stress and MeJA induction. Combined with these significant positive correlations, the results further identified *DoCHI* (LOC110104562) and *DoGTMT* (LOC110098370) as potential downstream targets of *DoGATA16* to regulate flavonoid biosynthesis to adapt to salt stress. However, this needs to be further confirmed through transgenic experiments in *D. officinale* in the future.

## 4. Materials and Methods

### 4.1. Plant Materials and Hormone Treatments

*D. officinale* seeds were cultivated in Murashige and Skoog (MS) medium including 2 mg·L^−1^ 6-benzyladenine (6-BA; Aladdin, Shanghai, China), 0.5 mg·L^−1^ 1-naphthaleneacetic acid (NAA; Aladdin), 20% fresh banana puree, and 0.7% agar [39] to generate seedlings in an environment of 25 °C with a constant photoperiod and a cold fluorescent white light intensity of 50 µmol m^−2^ s ^−1^. We collected 36-month-old *D. officinale* seedlings which were identified by Professor Shen Xiaoxia (Zhejiang Chinese Medicine University) to analyze gene expressions in different tissues (root, stem, leave, flower).

*D. officinale* seedlings (7–8 cm tall) grown in vitro were subjected to an MS medium supplemented with 250 mM NaCl (Aladdin) under the culture environment and the leaves and roots were picked at 0 h and 12 h. To investigate the effect of hormones on the expression level of GATA genes, the uniform growth *D. officinale* leaves were sprayed with 1 mM methyl jasmonate (MeJA, 98% purity, Sigma-Aldrich, St. Louis, MO, USA), which was initially solubilized with 2 mL absolute ethanol (99.5% purity, Merck, Darmstadt, Germany), and then diluted with distilled water. Plants sprayed with equal parts of distilled water supplemented as the control (CK). After 30 d of hormone treatments, we collected the three experimental samples. All experimental treatments were conducted with three independent biological replicates, and the collected samples were immediately frozen in liquid nitrogen and then stored at −80 °C for subsequent analysis.

### 4.2. Genome-Wide Identification of DoGATA Proteins

The 30 GATA proteins in *A. thaliana* recruited from the online website TAIR (https://www.arabidopsis.org/) (accessed on 4 September 2024) were utilized to query the genome of *D. officinale* to obtain GATA family genes. Hidden Markov Model (HMM) of GATA protein (PF00320) was downloaded from the Interpro database (https://www.ebi.ac.uk/interpro/) (accessed on 5 September 2024) and *D. officinale* GATA sequences with E-value > 0.05 were analyzed by TBtools v. 2.127. After verifying the integrity of the conserved structures of the proteins acquired by the above two methods in the NCBI-CDD database (http://www.ncbi.nlm.nih.gov/Structure/cdd/wrpsb.cgi) (accessed on 5 September 2024), 18 DoGATA proteins with complete sequences in the conserved structural domains were finally identified. Subsequently, they were named *DoGATA1–DoGATA18* according to the position on the chromosomes.

In addition, the physicochemical properties including amino acids number, molecular weight (MW), isoelectric point (pI), instability index, aliphatic index, and grand average of hydropathicity (GRAVY) were analyzed through ExPASy (https://www.expasy.org/) (accessed on 7 September 2024). The WoLF PSORT website (https://wolfpsort.hgc.jp/) (accessed on 7 September 2024) was used to speculate the subcellular localization of the DoGATA proteins.

### 4.3. Phylogenetic Analysis and Multiple Sequence Alignment of DoGATA Proteins

To understand the evolutionary relationship of DoGATAs, a total of 30 *A. thaliana* and 27 *O. sativa* GATA protein sequences were downloaded from PlantTFDB (https://planttfdb.gao-lab.org/) (accessed on 10 September 2024), as well as 18 *D. officinale* GATA protein sequences which were submitted to MEGA11 v. 11.0.13 to construct a neighbor-joining based evolutionary tree, and the bootstrap value was set to 1000. Meanwhile, a phylogenetic tree was visualized using Chiplot (https://www.chiplot.online/) (accessed on 15 September 2024). In addition, *D. officinale* GATA proteins were subjected to multiple sequence alignment using DNAMAN v. 9.0 (https://www.lynnon.com) (accessed on 22 September 2024).

### 4.4. Gene Structures, Conserved Motifs, and Cis-Acting Element of DoGATA Genes

The distribution of exons and introns of the *DoGATA*s were analyzed and visualized by the Gene Structure function of TBtools v. 2.127. The MEME (http://meme-suite.org/) (accessed on 25 September 2024) online website was used to identify the conserved motifs of the *DoGATA* family members. The analysis parameters were configured with an amino acid width range of 15 to 50 and the maximum motif number set to 10. The upstream 2000 bp sequences of *DoGATA1*–*DoGATA18* were retrieved from the genome of *D. officinale* and submitted to the PlantCARE platform (http://bioinformatics.psb.ugent.be/webtools/plantcare/html) (accessed on 30 September 2024) to predict the *cis*-acting elements responding to growth and development, hormones, and stress.

### 4.5. Chromosome Localization, Collinearity Analysis and Protein Structure of DoGATA Proteins

The location of 18 *DoGATA* family genes on chromosomes were visualized by Gene Location Visualize from GTF/GFF of TBtools v. 2.127. In order to explore duplication and mutation events in the *DoGATA*, collinearity analysis of the *DoGATA* was performed and visualized using the One Step MCScanX modulation.

*A. thaliana* (dicotyledonous model plant) and *O. sativa* (monocotyledonous mode plant) genome files and gene annotation files were downloaded from CNCD-NGDC (https://download.cncb.ac.cn/gwh/Genome/Plants/) (accessed on 9 October 2024). The collinearity analysis was also plotted by the Dual Synteny module in TBtools v. 2.127. The secondary structure of GATA proteins were predicted by the SOPMA (https://npsa-prabi.ibcp.fr/) (accessed on 15 October 2024) website, and the tertiary structure of proteins was predicted by SWISS-MODEL (https://swissmodel.expasy.org/interactive) (accessed on 17 October 2024).

### 4.6. RNA Extraction and Quantitative Real-Time PCR Analysis

The total RNA of different tissues (root, stems, leaf, flower), salt-treated leaf and root, and MeJA-treated leaf was extracted by Quick RNA Isolation Kit (Huayueyang Biotechnology Co., Beijing, China). RNA structural integrity was assessed through 1% agarose gel electrophoresis followed by precise quantification of concentration using a NanoDrop 2000 spectrophotometer (Thermo Fisher Scientific, Waltham, MA, USA). The Evo M-MLV RT Kit II (Accurate Biology, Hunan, China) was used to obtain first-strand cDNA for detecting the expression level of the genes.

Quantitative real-time PCR (qRT-PCR) primers for *DoGATA1*–*DoGATA18* genes were designed by Primer Premier 5.0 (https://sg.idtdna.com/PrimerQuest/Home/Index) (accessed on 20 October 2024) and generated in Zhejiang Shangya Biotechnology Co (Table S1). Elongation factor 1 alpha (EF-1α) was employed as a house-keeping gene [40]. The qRT-PCR implemented through 5 μL 2× iTaq™ universal SYBR^®^ Green (Bio-Rad Laboratories, Hercules, CA, USA), 1 μL cDNA, 0.4 μL of each primer (10 μM), and 3.2 μL RNase-free water were reacted on an Applied Biosystems (Applied Biosystems, Foster City, CA, USA). A 2^−∆∆Ct^ algorithm [41] was used to calculate the relative expressions of *DoGATA* genes.

### 4.7. Co-Expression Analysis Between the Enzyme-Encoding Genes and DoGATA Genes

Some studies have identified enzyme-encoding genes involved in the flavonoid metabolic pathway [42]. The correlations between the *DoGATA* genes and these enzyme-encoding genes was performed in SPSS v. 27.0 (IBM, Armonk, NY, USA) with Pearson’s correlation coefficients.

### 4.8. Subcellular Localization of DoGATA16

The coding sequence of *DoGATA16* (excluding the stop codon *TGA*) was amplified using PrimeSTAR Max Premix (Takara, Dalian, China) and cloned into the SpeI/BamHI sites of the pHB-YFP vector [43] containing the CaMV 35S promoter. The DNA sequences were verified in the Zhejiang Sunya Co. (Hangzhou, China).

The recombinant plasmid (pHB-*DoGATA16*-YFP) and the empty vector control (pHB-YFP) were transformed into *Agrobacterium tumefaciens* GV3101 (Weidi Biotech, Shanghai, China) using a freeze–thaw method [40]. The transformed *A*. *tumefaciens* were then infiltrated into leaves of 4-weeks-old *Nicotiana benthamiana*. After 48 h, YFP fluorescence was examined using a confocal microscope (Zeiss, Oberkochen, Germany) with a 488 nm.

### 4.9. Statistical Analysis

All experiments utilized in this study were performed with three independent replicates. The date presented as the mean ± standard deviation (SD) was calculated and performed in GraphPad Prism v. 8.0.2 (GraphPad Software, Boston, MA, USA). In all graphs, *** indicated extremely significant differences (*p* < 0.001), ** represented highly significant differences (*p* < 0.01), and * displayed a significant difference (*p* < 0.05).

## 5. Conclusions

In this study, we identified 18 *DoGATA* genes from *D. officinale* genome and comprehensively characterized them through phylogenetic, structural, chromosomal, and promoter analysis. Expression profiling across tissues and under treatments (salt and MeJA) revealed functional diversity, with *DoGATA16* emerging as a key regulator of flavonoid biosynthesis and stress response through co-expression analysis.

This study provides a basis for additional studies of *GATA* genes in other plants and sheds light on the molecular breeding of *D. officinale* in genes when integrating into a “stress-hormone-metabolism” regulatory network to reveal how *D. officinale* can be used to balance stress adaptation and secondary metabolism.

## Figures and Tables

**Table 1:** Physicochemical properties of DoGATA proteins. MW, molecular weight; pI, isoelectric point; II, instability index; AI, aliphatic index; GRAVY, grand average of hydropathicity.

Name	Size/aa	MW/kDa	pI	II	AI	GRAVY	Localization
DoGATA1	329	35.73	5.97	53.41	61.12	−0.621	Nucleus
DoGATA2	160	18.33	9.62	63.84	60.31	−0.846	Nucleus
DoGATA3	250	28.39	8.91	55.12	50.76	−0.856	Nucleus
DoGATA4	756	85.03	6.56	49.12	73.03	−0.430	Nucleus
DoGATA5	327	34.73	6.00	64.61	62.75	−0.382	Nucleus
DoGATA6	140	15.31	9.60	57.27	55.14	−0.729	Nucleus
DoGATA7	221	24.63	9.67	52.97	69.28	−0.571	Nucleus
DoGATA8	205	22.84	8.66	80.3	60.59	−0.774	Nucleus
DoGATA9	387	43.60	9.12	48.29	77.08	−0.422	Nucleus
DoGATA10	211	24.17	9.75	58.02	58.67	−0.833	Nucleus
DoGATA11	243	27.66	9.36	56.15	60.16	−0.822	Nucleus
DoGATA12	261	28.09	8.26	48.33	61.69	−0.544	Nucleus
DoGATA13	499	55.84	5.40	42.72	70.52	−0.712	Nucleus
DoGATA14	325	34.92	6.46	56.15	67.02	−0.352	Nucleus
DoGATA15	259	27.74	9.75	80.15	59.54	−0.537	Nucleus
DoGATA16	456	49.28	8.61	70.72	62.72	−0.493	Nucleus
DoGATA17	272	30.19	8.67	67.25	58.09	−0.724	Nucleus
DoGATA18	199	21.49	9.97	77.41	56.13	−0.706	Nucleus

**Table 2:** Prediction of the secondary structure of the GATA protein sequence in *D. officinale*.

Protein Name	α-Helix	β-Turn	Random Coil	Extended Strand
DoGATA1	25.84%	7.60%	55.93%	10.64%
DoGATA2	34.38%	10.00%	36.25%	19.38%
DoGATA3	22.40%	3.20%	65.22%	9.20%
DoGATA4	33.90%	9.66%	40.08%	16.27%
DoGATA5	22.63%	7.03%	55.60%	14.68%
DoGATA6	25.00%	5.00%	55.71%	14.29%
DoGATA7	31.22%	3.17%	60.63%	4.98%
DoGATA8	20.49%	5.85%	61.95%	11.71%
DoGATA9	25.06%	6.46%	51.94%	16.54%
DoGATA10	27.96%	7.58%	43.13%	21.33%
DoGATA11	21.81%	3.29%	62.96%	11.96%
DoGATA12	22.61%	5.75%	61.30%	10.34%
DoGATA13	31.26%	9.62%	63.75%	15.23%
DoGATA14	16.62%	3.08%	71.69%	8.63%
DoGATA15	13.90%	6.56%	64.09%	15.44%
DoGATA16	21.71%	3.95%	64.47%	9.87%
DoGATA17	14.71%	2.94%	74.26%	8.09%
DoGATA18	29.65%	7.04%	53.77%	9.55%

## Data Availability

All data generated or analyzed in this study are included in the main text and its Supplementary Materials.

## Supplementary Materials

The following supporting information can be downloaded at https://www.mdpi.com/article/10.3390/plants14111576/s1, Table S1: Primers used for qRT-PCR analysis of DoGATA genes. Table S2: Protein sequences of 18 DoGATA proteins. Table S3: Protein sequences of 26 OsGATA proteins. Table S4: Protein sequences of 30 AtGATA proteins.
