# Supplementary material for: Genome-Wide Identification and Analysis of GATA Gene Family in Dendrobium officinale Under Methyl Jasmonate and Salt Stress
Source: Plants (Basel). 2025 May 22;14(11):1576. doi: 10.3390/plants14111576 (PMC12157914; doi:10.3390/plants14111576)
Supplement: Supplementary file 1 [file plants-14-01576-s001.zip › plants-3570153-supplementary.pdf]

Supplementary Materials

Table legends

Table S1. Primers used for qRT-PCR analysis of *DoGATA* genes.

Table S2. Protein sequences of 18 DoGATA proteins.

Table S3. Protein sequences of 26 OsGATA proteins.

Table S4. Protein sequences of 30 AtGATA proteins.

Table S1. Primers used for qRT-PCR analysis of *DoGATA* genes.

Gene ID	Forward primer (5′ – 3′)	Reverse primer (5′ – 3′)
DoGATA1	CAGAGCTCGAATGGCTATCA	GGTTGAGAGGAGGTGGATTT
DoGATA2	GAGCCCGTTAAGTTCTGTTCT	CACTCTTCTCCTCTTGCTGTATC
DoGATA3	CAATGCTTGTGGTGTGAGATTC	GAGAGTTGGAGTGCTTGTAGTT
DoGATA4	CCAGTGAGATCCCAGAGAGATA	CGGATCGACAAGCTTCATTAGA
DoGATA5	GCTGGGTCTGTACAACGATATG	GTGGTGGAGTTGGGAATGAA
DoGATA6	GAGAATGATGGGCTTTGGTAGA	GAATGGCTGCTTCCTCTTCT
DoGATA7	GAAGGCGTCAGCAAATTCAAG	GTGCTGCAATCAGAGCAAAC
DoGATA8	AGGACCGAAGACTCTCTGTAA	GAATGTATAGCGGCGGAGAAA
DoGATA9	CTGTGTAATGCCTGCGGAATA	ACTGCACCACTGCTCATAAC
DoGATA10	AGGAGGAAGAGAGCAGAGAAA	TCTCCAAAGTGGCGTGTATG
DoGATA11	TGAATGCGGAGAAGGGTATTG	TCCACTGTGGTGTCCTATGT
DoGATA12	CCGCCGACATCTTCTACTAATC	AATTCCGCTCTTTCCTCTTCTC
DoGATA13	GCACTAAGCAACGGTCAAATC	CCGAACCGTATAGCGTATCTTC
DoGATA14	TGGGAGATGGAGTTTGGAATG	GAAGAGGTCATGGTGGCTAAA
DoGATA15	GGTCATGCTCTAGCTCTGTTAAA	GGATGATGATGAGGAAGGAGAAG
DoGATA16	CGTTCTGGTAAGTCGAGTCAAG	CCCACATTGGTTTCTGCTTTC
DoGATA17	TTTCAGCACCTTCTCTGTTTCT	CGCTTCTTCTGATAGGACTCTTG
DoGATA18	AACGAGGAGGAAGAAGAGGA	CGGAGCTGCTGCTAAATGA
EF-1α	TCAGGCTGACTGTGCTGTCCT	GTGGTGGCGTCCATCTTGTT

Table S2. Protein sequences of 18 DoGATA proteins.

Protein name	Amino acid sequences
DoGATA1	MEGCCHAGTGQLVPEKQGKVVGDHCIVEELLDFSNEAEGDTEMA<br>AEEDGVSEETGGNCSADSCANSSSGGGGASHTRFADELVCRSLAD<br>VGLSGDLCEPVLIFDPSKTLMREYEELAELEWLSKFVEDSFSSDEP<br>HKQQLISGLKSTSSQPTHGTNSHFPTATDAAHEPPPFTTDAPVPGK<br>ARTKRARTAPCSWSSRLLLVSLSSEKKKSPFPAALSPSPDGRKCLH<br>CAAEKTPQWRTGPMGPKTLCNACGVRFKSGRLVPEYRPAASPTFV<br>LSKHSNSHRKVLELRRQKETQDQQPQQFLTVHNRTTAAGDEFLIQ<br>HRLLEDGRRKFG
DoGATA2	MILGHGSVMMTRKRTKVDEQCKEKTATSMDEPVKFCSDCQTPNT<br>PLWRSGPDGPKSLCNACGIRYSKRRRVEFKRAEENKPTAMSCKNE<br>GKVAVEVEELKRRQREKQEKMLKVLLFRRKNLNVRWRKWPPAN<br>FGGNGDGEEVAEAARLLLCLSSEMSIHS
DoGATA3	MDVYGKAKVPGEVSTDFQSEMLSSPAGAIWEDFIPGQNMERDEE<br>DNISLQWLSFFMEDCFSSTTSFNFPTPLIPTKTNNASNSCATDPYPA<br>PLRNPNPCSFQKPSFPAKARNKRRKTKTSSQFINSSIDSPLLHQKH<br>WLAESELLISPKMEKNENKPETEDEEKGINGRGLQHQQPKRCSHC<br>LSHRTPQWRTGPLGPKTLCNACGVRFKSGRLLPEYRPAKSPTFVN<br>YKHSNSHKKVMEMRMADFSTNYI
DoGATA4	MFEGYSRELASAISTSEIPERYFRPEAEARVVDSIDDDELPVIDLMK<br>LVDPELFDKEELSRLGLACREWGFFQLINHGVTKEMIEKTKNDIM<br>GFFKLPLKEKEAFKQLPGQIEGYGQMFVHSEEQKLDWADMLTLV<br>TKPHKDMRFWPINPSSFRDTIDKYTVKIENVVDCLYALMAKDLGI<br>DPKVMLKNLSKQMQSIRINYYPPCKEANKVLGLSPHSDGIALTVL<br>LQANDVNGLQIKKNNKWLLVKPKPGAFIVNIGDMLEIMTNGKYK<br>SVEHRAIINMEEARLSIATFHLPNTDVILRPFPELISGDGELYKTISV<br>KEYTKDFLEFLSSYRDHVYYWHRMMVERISMFCADLKFSALTAH<br>LTSIQDHRERVVSRRTWRVGQLAIAVSSKWRGPGHAHTVRLRAVR<br>RLMWWPLTAPPTSRRFFPAGRLKRHPSYEPHFMDGVSNWEASVH<br>QNIYHIGGDERQTRRADFGMGKGMEMTASPATFAGASPAYMYSM<br>PNAESLRVDELLDFTNHDLFPSSSSSSSSVPIDEAALHLPAGQEFSSS<br>ASVAATVGFDASLPDDICIPSEEAAELEWLSKFMEDSFSDTPYHYS<br>LPDMTMSENSNYPSSEISTHALASGINRSPEISTSSSSSSSSSSSDFPK<br>AKTNVINKKKQGNNESGARRCTHCSSEKTPQWRTGPLGPKTLCN<br>ACGVRFKSGRLVPEYRPAASPTFVLTQHSNSHRKVMELRRQKEFLI<br>LRGSGGGGGDGGSSGFSTEMSFQDYEVC
DoGATA5	MAMKAFMEPMGTELVCEDPFDSIDDLLDFPNDDDALPMAEAWG<br>DCAPLAPSPAPAPSLMAAGSGGGISVGSDASGLDGRAAAVSGVGIS<br>SENDALGLYNDMDIANLEWLSNFFEDSEFNFDFIPNSTTPSTPAAK

	PENKTASLSRSSSPISVLLPTTSSSSSSSSSSNSITDISPPPPPPASVPGRARTKRPRLPSFSARTFIPPSSNTPISSPAATKKMKRDLDSVPSMRKCSHCDIQKTPQWRAGPMGPKTLCNACGVRYKSGRLFPEYRPAASPTFVPAVHSNSHKKVVEMRLRCTEMVGKSGGREIPVAAKNCDLLEYIRRRD
DoGATA6	MDLSGEKATGSTDPSADTSNRDCEVSIFDGSKSCTDCGTTKTPLWRGGPSGPKSLCNACGIRYRKKRRAAIGSTKERSEDTRNNSSSKKMSSLKMRMMGFGREIIWKPGSLVRNQKNRRLGEEEEAAILLMAISSGFIYA
DoGATA7	MERQLEIKESSDDQYSSSCFYEPAGEGHQHESSVKWMSSKMRIMRKLMCSDQNNTVRKVERRRQQIQDDQKKKGNKDQSSCGSSNNGGISRVCSDCSTTKTPLWRSGPLGPKSLCNACGIKQRKARKAMATALTAVSGKLQPVVTIKPRMNKALKVDCSVPFKKRCRFFTSAETSQRTLSFEDIVISLNKSLLALNRVFPQDEKDAAILLMALSSGIVCSL
DoGATA8	MIDLPAAAARATAEAEAEAFPDDVLLLDFSSDPNCNLDAPTPQLFQEIFDAAIDDPGSAVDGEEEELEWLANKDAFPPLETSFDPVSRLPFRPPSRARSGGRRRRRKALPTFPPSTAPEKKQCRHCKAEETPQWRLGPEGPKTLCNACGVRYNSGRLVPEYRPANSPTFSAAIHSNSHRRVMEIRRRKYGGRRGRPRVRLVSESS
DoGATA9	MMMLVGLVHKFQDDCLQIEAFLGQEGMTRIEYLPNKSFWNPTRSKLYEFKITRKKLQVLDYTDGGMQICEIDISYIRPIGRGYKSCRTKELFFTEREMQCPRELNPLKSFQLQITINGYIKEVDQQQGPVKWMSSKMRIMRKMLNSNQTPKIKTRRNNHQHQDQKQAAQDNIIRVCSDCSTTKTPLWRSGPHGPKSLCNACGIRQRKARRAMAASVMSSGAVLKDGKDKASDFVDRTVPFKKRCRFATEGCETTQRKLCFEDIVVSLNKSSANFHRVFPQDEKDAAILLMALSCASVLTPMETPSPVENIPLKSPVLIPPTPVQMPVESSANVINVDPVETSANVAIAADLVQVIDHDKDSGLPNLNLPQTESSSAVYGASSWNTIF
DoGATA10	MPKKNPSSMICSTKYGKCACCAYDSSNSSASSDGRRRFVVTSRTSKEEESRERQVKTVVDSSIKVCSDCRTSYTPLWRNGPNGPMSLCNACGIRYRKRKISVRMEERKARKVFAITGNAESKRNSNAEETKKGQRVKREKMVMTLSEEEMRMRQRDKQEKILMVQFHLRREAGSWTQRALRERSDEGVESEEVAEAALLLLYLSCGISVRF
DoGATA11	MVVYRNTVVCGDIPAEFQPEKVPSSSNTMWEHFFPGQNKEEDEENDSLQWLSIYVEDCLSGNTSFTSPPSPTHSNTIKISQNPKQTQKLSSSFENFSLSVPAKARTKRRKTKDDSNFLNYSKESPLLHQKHWLAESELLIIPKMEKLDKLQVNAEKGIGNGRELQQLQPRRCSHCLSHRTPQWRAGPLGPKTLCNACGVRFKSGRLHPEYRPAKSPTFVSYMHSNS

	HKKVMEMRMAGLSSHSK
DoGATA12	MSENPSSPVAEEPMAGGMEGLHESAGSLVSIDGDATTDHGVSIVP HVGGSQLTLSFQGEVYVFDCVSPEKVQSVLLLLGGREVAAPLPSPP TSSTNQLNKRFNFSHRVASLMRFREKRKERNFDKKIRYDVRKEVA LRMQRNKGQFTSSKSKAEGTLPGFAGMDSTSYLAAENGQPQTAS ACYHCHISAKDTPMMRRGPDGPRTLCNACGLMWANKGTLRDLS KSPVTFQHQTLESKDGEGATATGQPTAAVPSNGQEISH
DoGATA13	MNQNSNPAVEVKLVAAGKYPPGCRGQDSIAARQVLASMTMPGQI DGGDVGVSSDGQGILHYTREHDENNCGGVENGMDEDQNGMME SEGMEGDGPSDPGHPCDPQGMIVPHGGSNQLTLSFQGEVYVFDSV SPEKVQAVLLLLGGREISNSLAAFPSTADLSTKQRSNLPHRVASLM RFREKRKERNFDKKIRYTVRKEVALRMQRSRGQFVSSKSKTEVVT SDVINWPNQHWDIVEKQPPSAVECHHCGISAKSTPMMRRGPNGP RTLCNACGLVWANKLHSKRSSKLGHGRLKDLVHVKYNRALIRRF DHRDVIDPISLIEIDDANEWLIEKLDDESDEENDFIAGDEDLTWTSV AKAIGVYDKSYNIRERSKGKEKESSIRHDIAIENEDEEGDEEDYEV GDNVDEDNEAFLTFADDDDSQEDLRYVQNGARHAMRYAPWLQR DLCAIVHHSPLTGMAIADNMHKAYLPCCEHRDEFPPIVLRKVRIG GCSPRCNR
DoGATA14	MARQWEMEFGMGVGMPAGFGSASAPAYIYGVPAAAETTLRVDEL LDFSHHDLFASSSSSSSVAAEANLVPAAQFSAGHVSNPEYHYPRHA SFADDIYIPVSSLLNKSDEVAELEWLSKFVEDSFSDVPVQSLAAET ALPNATAHRADVSNVVRGARSKRSRGVLTVVESEANHHAASAWS SLTPSSSTTSSSSSSDFPTTKGSSRKKLATGETVEDGGGVRQCTHCA SEKTPQWRTGPLGPKTLCNACGVRFKSGRLVPEYRPAASPTFVLT QHSNSHRKVMELRRQKELLILRRHDNASPSATFPSASIASAGTEMI YQDYNVY
DoGATA15	MQETDAASLEWLSRFCDDDCSKEFPSPSPPPSHLGMLPSLPTQIPL SKYSSAGSQTLAPVPVPALYSTATAAGSLSTEALVPVKAKRSKRLR GSGWSCSSSVKLSDSSPTSSITTLRTASPSPSSSSSSSSFNSYISYEPP PFTGFLPDDSSAPPKPQKPKKRGRKPKNPIISPAERRCTHCGVHKTP QWRAGPQGAKTLCNACGVRFKSGRLLPEYRPACSPTFVSNIHSNS HRKVLEMRRKKDDLVLVSSAPPVSTC
DoGATA16	MLHQTLVSFPFSSSSVSPCTIPLSSASAGSASSALPLFAGRLVSPPPP YPRSSQVEMTTIEDMLASARDQGFLPDEVEDLLWSADRGNPLGEE FQVEDLLDLRGLPESEEDEEAPRVTEASFSPPKPSPCVDNEPLGLYT EITFALAETDAASLEWLSRFCDDDCSKEFPSPSPPPSHLGMLPSLPT QIPLSKYSSAGSQTLAPVPVPSLYSTATAAGSLSTEALVPVKAKRSK RLRGSGWSCSSSVKLSDSSPTSSITTLRTTSPSPSSSSSSSSFNSYISY

	EPPPFTGFLPDDSSAPPKPQKPKKRGRKPKNPIISPAERRCTHCGVHKTPQWRAGPQGAKTLCNACGVRFKSGRLLPEYRPACSPTFVSNIHSNSHRKVLEMRRKKDDQRGRNAVRSGKSSQAHDVAIRSTLRATPICYEFAKKAETNVGFSSFPTSDLHFCPISSTYSRYCR
DoGATA17	MNGLNETISSVEEAPVVEEFQVDDLLDLRGLPEPEKNEEEDDGDAKGVAFVSFKKESPPLSGISLPEETDAADLEWLSRFVHDCHSEYQPAFASLKEKETACFPTKLPSPVDVWRLKKEALVPIKAKRSKRHRRTGAFWFIFSTFSVSSNTSSPSSSSSCSSSYKSQESYQKKRGRKPNNSAASGVSGDRQCTHCGVQKTPQWRAGPHGPKTLCNACGVRFKSGRLLPEYRPACSPTFVDNIHSNKHRKVLEMRRKNEGQLFSAAAQPVQSC
DoGATA18	MEAEGLAAQEGKNEEEEEELEWLANKDVFPALETSFDLSAQSFSSSSAASGDRRSPVSVLASALPFRAPAKLRTKGQRRRRKTLPCFIPPPPAAHPAASAGKRKCRHCESEETPQWRAGPEGPKTLCNACGVRFKSGRLVPEYRPANSPTFTASVHSNSHRRIVEMRRQKYGAAASSAAGGHGRGRRSLIAGLAKSAHT

Table S3. Protein sequences of 26 OsGATA proteins.

Protein name	Amino acid sequences
OsGATA1	MGSTDRKVVGIGVAEEGRRSCVECRATTTPMWRSGPTGPRSLCN ACGIRYRKKRRQDLGL DLNQPQKQEHGEVIPEVKDSNSNSNNCNSGSGNSSSNLQVVPKRR LLMGVEEAALLLMTL SSPSASTLLHG
OsGATA2	MLHEAAPCTCGLLYGSCGGGCSLLFPAGAPGDHHHHHHYKQYCG AGDGEYPDVPYGGGGSVDCTLSLGTPSTRRAEAAVAGLPWDQSS LQPSCNGRQEMSGAAAPRTEPSGGAGAAAASAPRRCANCDTTST PLWRNGPRGPKSLCNACGIRYKKEERRAAAAAVAPTALASDGGVE YAYGYPRQQQQWGCYGPAVAKAASFGMFGDAAGEDGPCLPWGL GVMPSSPAFGAVREMPSLFQYY
OsGATA3	MEVTAEFGGAYYGGAAGREKKALQQGCGDHFAVDDLLVLPYGE EDETTREGEATGGKEEAAGFGNASADSSTITALDSCSNSFGLADG DFPGELCEPYDQLAELEWLSNYMNEGDDAFATEDLQKLQLISGIPS GGFSTASVPSAQAQAASAAASMAVQPGGFLPEAPVPAKARSKRSR AAPGNWSSRLLVLPPPPASPPSPASMAISPAESGVSAHAFPIKKPSK PAKKKDAPAPPAQAQLSSVPVHSGGSAPAAAAGEGRRCLHCETDK TPQWRTGPMGPKTLCNACGVRYKSGRLVPEYRPAASPTFMVSKH SNSHRKVLELRRQKEMHQQTPHHHQPQVAAAGGVGSLMHMQSS MLFDGVSPVVSGDDFLIHHHLRTDFRPPI
OsGATA4	MDMDSSSSPVDKVDPDECNGSKACADCHTTKTPLWRGGPGGPKS LCNACGIRYRKRRRAALGLDSSATATATDGAEQQKKTKAKKEKA QEEEVTMELHTVGFRSKDAAVFKQRRRMRRRKCLGEEERAAILL MALSSGVIYA
OsGATA5	MSGHHEAKPYQPRRGPAPADEEAAPAAAADEAEAEAEVEAMERY EQEQEYEEGEEGEEEEYEGGEGVPMDADASAAAVAGMDPHGEM VPVAGGEAGGGYPHVASNTLTLSFQGEVYVFESVSAERVQAVLLL LGGRELAPGSGSVPSSSAAYSKKMNFPHRMASLMRFREKRKERN FDKKIRYTVRKEVALRMQRNRGQFTSSKSKAEEATSVITSSEGSPN WGAVEGRPPSAAECHHCGISAASTPMMRRGPDGPRTLCNACGLM WANKGTMREVTKGPPVPLQIVPAATNDVQNGIVEATGVEQHNSA VEEAVSAANGHESQSGVA
OsGATA6	MSTIYMSQLPATLPLMEGDQDQGLYPAFHRAKDPPILFPFMIDSAV EHQGQIYGDQGLRR QQVLGESNQQFNDHMMMGGSDVFLTPSPFRPTIQSIGSDMIQRSS YDPYDIESNNKQHAN GSTSKWMSTPPMKMRIIRKGAATDPEGGAVRKPRRRAQAHQDES

	QQQLQQALGVVRVCSDCNTTKTPLWRSGPCGPKSLCNACGIRQR<br>KARRAMAAAANGGAAVAPAKSVAAAPVNNKPAAKKEKRAADV<br>DRSLPFKKRCKMVDHVAAAVAATKPTAAGEVVAAAPKDQDHVIV<br>VGGENAAATSMPAQNPISKAAATAAAAAASPAFFHGLPRDEITDA<br>AMLLMTLSCGLVHS
OsGATA7	MTHQALIPSTPPSAFSPASHFLHASSSSSPSLSSHAVVATAAAAMSS<br>FAHHHHGSLVEKDGRMSALRSSLRPYEAAEEMAAAAAAGGPAA<br>AWGAVERGAGMMGDGFSVEDLLDLEELCEVDRDGGEQGEAAAA<br>AAAAVEKERSSDSHGSSVVSYEPMPLLPPVMDLPAHDVEELEWVS<br>RIMDDSLAELPLPQLPAAAAALAACGKPQHRRPHEGAASALLDP<br>MRTPTICALSTEALVPVKSRRSKRSRASVWSLSGAPLSDSTSSSSTA<br>TTSSCSSSASFSPFLQYVDFPALVASDLLDEQPRSKKSKHGKNGKQ<br>KPKKRGRKPKHQQPPHLAAAAGGGAALPATGDRRCSHCGVQKTP<br>QWRAGPEGAKTLCNACGVRYKSGRLLPEYRPACSPTFVSSLHSNS<br>HRKVLEMRRKKETPVIVAAAAPAVASF
OsGATA8	MAGVGFVEDMLREQSLLEATCGDLFDHIDDLLDFPKEESAADVLL<br>LDAPAPGSPLSSRIIGGHATMAAAPPPPPQMMALPPPPAPAKDDAS<br>ALFDAAGALGAEVFDRKDAHIGPCDELDMDMAQLEWLSGLFDD<br>GTIPHEPSFPGVNCAAPIKASALTANAGVVLPDKAEEALFRSSSPI<br>SVLEHSGFNVATNGGSSSSSSSSASSSSESFSGSGRAWSAPVSPRPE<br>PPVLVIPARARSKRSRPSAFPAVRGAPAATETTILVPTPMYSSTSSHS<br>DPESIAESNPHPPPMKKKKKAKKPAAPAAASDAEADADAADADY<br>EEGGALALPPGTVRRCTHCQIEKTPQWRAGPLGPKTLCNACGVR<br>YKSGRLFPEYRPAASPTFMPSIHSNSHKKVVEMRQKATRTADPSC<br>DLLQYIRRRD
OsGATA9	MRKPTPYVSLHDVVAFDFVDGDVPFDDLVDGEGLCCPDDPFEEV<br>MRCLSAVDDPFLAAFKLDCSPPTPAADADVDSRSEEHMHADVGG<br>GLDLQRAVGGGDEKAGTPSTVDDVPWLQASAVARKPRRAPAAVR<br>KRVWSLVSPQLATAAAAAVDNSRDEVSSGGGGGGEGGEHCSRPA<br>KRRRKCGEEKRCGHCQTTETPQWRVGPDGPSTLCNACGIRYRIDH<br>LLPEYRPSTSPGFGSDGYSNRHRKVVKLREKKRKKAMLAATATAL<br>TSGPV
OsGATA10	MVGDKDAAALAGELTGDAGASLNGFFDHTGLESAVVGEGQGEG<br>EEEEELEWLSNKDAFPSVDTMAAEVESAAPGAPARAAVGPRTKG<br>LRRRRRVTAPWSLAPLLSRPRQAAAAAADAGAPRRRCTHCAVDE<br>TPQWRLGPDGPRTLCNACGVRFKSGRLFPEYRPANSPTFSPLLHSN<br>SHRRVMEMRLQSEEDASAASRVNAKARRAERAAARLAGKDKK
OsGATA11	MPDAAAAAAAAQDADAVMRDAPADAAAGGGDNDDDDGDDGT<br>EEDEEEDDDEEGDEEELPPAEDPAAPEPVSALLPGSPNQLTLLFQG

	EVYVFESVTPEKVQAVLLLLGRSEMPPGLANMVLPNQRENRGYDDLLQRTDIPAKRVASLIRFREKRKERNFDKKIRYAVRKEVALRMQRRKGQFAGRANMEGESLSPGCELASQGSGQDFLSRESKCQNCGTSEKMTPAMRRGPAGPRTLCNACGLMWANKGTLRNCPKAKVESSVVATEQSNAAVSPSGIDNKELVVPNPENITASHGEVMGDSTPANEAEIGAPKAQSQ
OsGATA12	MAAEPPADGRDPPADDGAAGDGAVESAAAEALLSAASEQLTLVYQGEVYVFDPVPPQKVQAVLLVLGGSDMPPGLVSMAVPTTFDEKSTTVAARRVASLMRFREKRKERCFDKKIRYSVRKEVAQKMKRRKGQFAGRADFGDGSCSSAPCGSTANGEDDHIRETHCQNCGISSRLTPAMRRGPAGPRSLCNACGLMWANKGTLRSPLNAPKMTVQHPADLSKTGDTDDSKANLCAEHNQTTMKTDTEMVPEQEQKADVLPPTKEEDSMATS
OsGATA13	MDSSSVEKGSGSIDPDERTASGEPKACTDCHTTKTPLWRGGPSGPKSLCNACGIRYRKKRREALGLDAGEGGAERQEKKKSKRERGEEVTMELRMVGFGKEVVLKQRRRMRRRRRLGEEEKAAILLMALSSGVIYA
OsGATA14	MDALKSSCRSEEAADEGAAAAPSAWGMVERDGFSVEDLLDLEEFCEAEKDAAEENEQALALVAAPEEEKSKDDSQPSSVVTYELVAPPPPPPEIVDLPAHDVEELEWVSRIMDDSLSELPPPPQPPASVVASLAARPPQPRQLQRRPQDGAYRALPPASYPVRTPTICALSTEALVPVKAKRSKRSRATAWSLSGAPPFSDSTSSSSTTTTSSCSSSASFSSFSPLLKFEWHPLGGTSDLPDDHLLPPGKKSKHGKNGKNKPKKRGRKPKQLPPHPSGAAASAPAPGDRRCSHCGVQKTPQWRAGPEGAKTLCNACGVRYKSGRLLPEYRPACSPTFVSAIHSNSHRKVLEMRRKKEVGSGLLTAAAAAAPAVASF
OsGATA15	MGKQGPCRHCGVTSTPLWRNGPPDKPVLCNACGSRWRTKGSLTNYTPMHARDDIDAEEPRASKLKPPTLKLKEQKQLKKNPSHITMENGPFSDQNFRKMGDPDLSNRSGSGSALSYSESCAPYGTADASEMTASAQSHAWESLVPSKRRSCVTRPKPSQMEKLAKDLNSIMHEEQLLYLSGSSEEDLIYHSATPVDSFEMGYGSMLLRPNSKSLEEESEASSIPADNKSYITSESYSGSVSFVYSESKATSNQNVITEQPKKFLVQTSDNARRANLHTENQDTLENANSPLVSLHMEGKDSEETRVKTSASNRLTKSTMNPLKRPHDTHFQSSVELRGTMRSPKRVSKYGDAMGLKCQASFMPKPGNGKDLACSDRALNLFMLPPDKLSMLVPPQYANTDSDQDLLLDVPLNARHPEAELLCQPSQLSSVAHSSTSEAGNAGGEGRLKQP

OsGATA16	MGSSDQKVIGIAAAAAAAAEEAGRRCCVECGATTTPMWRGGPTG<br>PRSLCNACGIRYRKKRRQELGLDKKQQQEHHPHHHQQQQQQYQ<br>RQQQQQQQEDHSDAASSVKDSSSSSSNKSSSLQVISEIVISRSKSDC<br>EGAMEGNCVPLKRLVQQVDFLLSSTGITESCQCVAVSCANQMGL<br>QKAANVLLFLVPIRVLTMENNSCDILHIIRIIGRGCGIESKTRIIDR
OsGATA17	MEVAAADYAGGVRVKKEAGGCGGSGDMFLVDDLLDLPCDEEEE<br>ETGLCGAYGGGGAGLGAGVVGGGGDDRAAGNASADSSTVTAVD<br>SCSNSFSGLADGDFSGGLCEPYEQLAELEWVSTYMGEETLPTEDL<br>RKLQLISGIPAAPRAPPALAVSAVQLPAGGAGALPTEAPVPGKARS<br>KRSRVAPCSWSSRLMVLPPPPASPPSPASAVISPSESGTAAPAFPAKK<br>AAKSAKKKDGPSPAPAPNAAAQAAAEGRRCLHCETDKTPQWRTG<br>PMGPKTLCNACGVRYKSGRLVPEYRPAASPTFVVSKHSNSHRKV<br>VELRRQKEMQLLHHHQQPPPHVGAGGGGAAGGLLHVTSPLLFD<br>GPTSSAPLFAGADEFLIHNRISPDYRRQAT
OsGATA18	MLQELAPCTCGMLYGSCGGGCGGAAAAASAFSLLFPMAGGQYY<br>YRQCGGVAEEDSRSPYGGGGAAVDCTLSLGTPSTRRAEAGAYGG<br>GLQPWDVPSSARPGGGGGGKQDGAGVAPCNKEAPAAGRLPRRC<br>ANCDTMSTPLWRNGPRGPKSLCNACGIRYKKEERRAAAAVAPTPP<br>PSLDTGAGYAYCYSRQPPPPPAPQWGCYGQAAAKSASYAMFDAA<br>DDGPCLSWRLNMMPSSPAFAVGERPGLFQYY
OsGATA19	MLHHYYSGGAGHHQDVAAAGSPGDMASSTFSLFFPMSNGQCWP<br>PSTVEESAAYDDHSTVTTSPSSPSSSSTGSVDCTLSLGTPSSRRAEP<br>VAAAAPAANHGAPVPAHYPSLSAATVSWDATAESYYCGQQGRPA<br>TGAAKCAAGAGHDALLDRRCANCGTASTPLWRNGPRGPKSLCN<br>ACGIRYKKEERRAAATTTTADGAAGCGFITAQRGRGSTAAKAAPA<br>VTTCGEETSPYVVGGGGGGGEVADAAYLAWRLNVVPPAATATAF<br>SVWPERASLYHYN
OsGATA20	MSTIYMSQLSAALPLMEGEHHHHHQDHHQGHFQAFSLQPKDPPV<br>LFPFVISRRSSSSSPSDSTTLSYGSDHHLTQQQQHQHQAMLEPQNM<br>IGGSSAGIFATPFPTVKSIRDDMIERSQFDPYDTEKLQASCGLAKVV<br>AGGKWSAVPAAKMKITRKMGEPSSGVTGGAATTVAPKKPRRRP<br>AQAYEDHGHGGAMGQAFGVIRVCSDCNTTKTPLWRSGPCGPKSL<br>CNACGIRQRKARRAMMASGLPASPNAAGPKAAAHSGAAAVAAA<br>QPKVKKEKRADVDRSSLPFKKRCKVVQVEDHQTLPAATNAAAAA<br>AMEETAESATVAPPPAPTTRGGTLVDSIGLSWSKTHAAATASCSFR<br>PSPVAPGFAAAVQDEITDAAMLLMTLSCGLVRS
OsGATA21	MARFEEEHRALGAEEEYEEEEDELEEEEEEMEEDEDAQHHEGVG<br>GEVAVPMDAEAAAQLDPHGGMLAASGAVQPMASNQLTLSFQGE<br>VYVFDSVSPDKVQAVLLLLGGRELNPGLGSGASSSAPYSKRLNFP

	HRVASLMRFREKRKERNFDKKIRYSVRKEVALRMQRNRGQFTSSK<br>PKGDEATSELTASDGSPNWGSVEGRPPSAAECHHCGINAKATPMM<br>RRGPDGPRTLCNACGLMWANKGMLRDLSKAPPTPIQVVASVNDG<br>NGSAAAPTTEQEIPAPATVNGHESST
OsGATA22	MVVVDGLHDGGGGDLQALLDDAGVDDVAARGGGEVEEEVERPS<br>NEDAFPAVEKMATAAAAKGLQCRHCGTTETPQWRHGPEGHRTLC<br>NACSMRYRSGKLVPEYRPLRSPTFSPELHSNRHHRVLQLRRRPGP<br>QSAAPSPAAVARYGGEAKEEEEELAWVSNKDAFATVETTMAPSPR<br>VVETPPEHDHRPANTPTTSPEPHSDRPRRVVQLPRRLQEPSASANL<br>AHAVAATARAGRECAHCGTTKTPAWRLGPDSRRKLCNACGNKYR<br>SGQLNSTTFSQNSQEQKKKSKSSACSRERKRSAVAATVVVGGGLR<br>DDAAAIADEHLDGGDLQALLDDVALDDVAARGGGDAGEAKEEE<br>EELEWLSNKDAFPTVETMSPAPPENRTKAPVPPAGWQCRHCGSTE<br>TPLWRERDGPAEAEHVRKEETPPNITPATKHRRIVDLLRCSTALNT<br>AAKAVERRCTHCGTTKTPAWLSGPDSRGKLCNACGKQYRKGRLV<br>PEYRPLNCPTFSPELHSNAHAHRRRRESPVAIAIAGEK
OsGATA23	MASEWEMAMGVDLGMGMSTYHNASGGIAAAPMMGHHGGGGG<br>GGGYSAAHHHHHHYYGMPHQAAMGDAMRVDDLLDLSNTPGAH<br>DFFPASAAAAAAGDHGHHHHHHIGGMGEPSGATPSATSSDHQTS<br>MLSFADDFYIPTEDAAELEWLSKFVDDSYSDMPNYQSSAHAAMA<br>AAAASAANNGGGSSAGQDSCLTAAPGRGARSKRSRATAAAAAA<br>WHSLVPRPPSQSSPSSSCSSSDFPSSNKPSGTARPNGSGGGSRGKKS<br>PGPAGAEVGMEAGVRRCTHCASEKTPQWRTGPLGPKTLCNACGV<br>RFKSGRLMPEYRPAASPTFVLTQHSNSHRKVMELRRQKELLIIRGS<br>HRDAAAAAAAAAAAAAAGSAAATGRPELMFRDYGVC
OsGATA24	MPKPTPSSSSFLDFTGGVDGDDDDPSCPFEGLCCPDDPLDQVLNF<br>DSSDFGHVFFESLDVELFLPRGGPSRGAGEEDSKGAVERVAFGSSA<br>AVESELGGVGGGGAGSEVSVPGGAGGGRGEDMETEALDVKPVV<br>GVGAGGAMGAHVAGGVGAPGAFPESKQLVPWPCAVGAGASAPG<br>AAPDNRLLALPDVRFDALTAEGAAPGGERGKTIPDSVSKNGLPTL<br>PGVRSATPTAPPATPFRLEWDHAAAPSSSATTTPSDSSLSSPPSLSSV<br>FPRIARVFPSRTKPRRRRTLRRQHWSLICPLHLVPVAAAADAARGK<br>SISELNASASAATDAGTPSINDGGGGSYHRRVVGRQRNRQVRKD<br>RRCSHCGTSETPQWRMGPDGPGTLCNACGIRSKMDRLLPEYRPST<br>SPSFNGDEHSNRHRKVLKLREKKGRD
OsGATA25	MDGDGDVGGGGGGGGGGGVRYVLALPAMASLAVLIAHLDAAVP<br>VPRRPRSYLPRAVPMAWWAFRLPVFRPPPPPPPPAKNPVKEEEGV<br>ARVVVVVAPPPPVDPGEEEAGKRAAKRARRCLNCDAVETPQWRS<br>GPMGRSTLCNACGVRLRAVGSLPEHRAPAARTTTAAPASPPDSPIW<br>TPGHKPPSSSPDIYLVRRTPKLPVTRPPRTKQAPPTAPAPAPPPPPPQ

	PASPKTKTKAKAKKPKRKRSCVHCGSTETPQWREGPTGRGTLCN<br>ACGVRYRQGRLLPEYRPKGSPTFSPSVHAANHRQVLELRRQQRQS<br>TNPSTPPPPPVSAAEPIPDEQKEEVVSVPVAAAAPATDGGAASSLD<br>ALLLDGPSAPLIVDGDDFLVS
OsGATA26	MDVHPPNAAASSLEELFPHQPATESDRSGIEWLSVYVEDCLSTSAS<br>CTNPVSAELPPITMASQGAAKPKLPPRSSTNARKKKRSLASVISDT<br>DDQHCITLFVEPPLLLLDHKDWLAESELILPKKDKDEELVQEQEQE<br>EEENYKMSAGMQFQQEQLVITCSYCLSSQSPQWWDGPSGPTCDA<br>CRLRIEARNGHTTSSKKRYGQEIDKEQDIGKRRDKKKIKKAVYVN<br>DELLSEEPMKRCTHCLSYKTPQWRTGPLGPKTLCNACGVRFKSG<br>RLLPEYRPANSPTFVSDIHSNSHKKVMQLRNSVPHPRK

Table S4. Protein sequences of 30 AtGATA proteins.

Protein name	Amino acid sequences
AtGATA1	MEMESFMDDLLNFSVPEEEEDDDEHTQPPRNITRRKTGLRPTDSF<br>GLFNTDDLGVVEEEDLEWISNKNAFPVIETFVGVLPSEHFPITSLLE<br>REATEVKQLSPVSVLETSSHSSTTTTSNSSGGSNGSTAVATTTTTPTI<br>MSCCVGFKAPAKARSKRRRTGRRDLRVLWTGNEQGGIQKKKTMT<br>VAAAALIMGRKCQHCGAEKTPQWRAGPAGPKTLCNACGVRYKS<br>GRLVPEYRPANSPTFTAELHSNSHRKIVEMRKQYQSGDGDGDRKD<br>CG
AtGATA2	MDVYGLSSPDLLRIDDLLDFSNEDIFSASSSGGSTAATSSSSFPPPQ<br>NPSFHHHHLPSSADHHSFLHDICVPSDDAAHLEWLSQFVDDSFAD<br>FPANPLGGTMTSVKTETSFPGKPRSKRSRAPAPFAGTWSPMPLESE<br>HQQLHSAAKFKPKKEQSGGGGGGGGRHQSSSSETTEGGGMRRCT<br>HCASEKTPQWRTGPLGPKTLCNACGVRFKSGRLVPEYRPASSPTF<br>VLTQHSNSHRKVMELRRQKEVMRQPQQVQLHHHHHPF
AtGATA3	MELWTEARALKASLRGESTISLKHHQVIVSEDLSRTSSLPEDFSVE<br>CFLDFSEGQKEEEEEVVSVSSSQEQEEQEHDCVFSSQPCIFDQLPSL<br>PDEDVEELEWVSRVVDDCSSPEVSLLLTQTHKTKPSFSRIPVKPRT<br>KRSRNSLTGSRVWPLVSTNHQHAATEQLRKKKQETVLVFQRRCSH<br>CGTNNTPQWRTGPVGPKTLCNACGVRFKSGRLCPEYRPADSPTFS<br>NEIHSNLHRKVLELRKSKELGEETGEASTKSDPVKFGSKW
AtGATA4	MDVYGMSSPDLLRIDDLLDFSNDEIFSSSSTVTSSAASSAASSENPF<br>SFPSSTYTSPTLLTDFTHDLCVPSDDAAHLEWLSRFVDDSFSDFPA<br>NPLTMTVRPEISFTGKPRSRRSRAPAPSVAGTWAPMSESELCHSVA<br>KPKPKKVYNAESVTADGARRCTHCASEKTPQWRTGPLGPKTLCN<br>ACGVRYKSGRLVPEYRPASSPTFVLTQHSNSHRKVMELRRQKEQQ<br>ESCVRIPPFQPQ
AtGATA5	MEQAALKSSVRKEMALKTTSPVYEEFLAVTTAQNGFSVDDFSVD<br>DLLDLSNDDVFADEETDLKAQHEMVRVSSEEPNDDGDALRRSSD<br>FSGCDDFGSLPTSELSLPADDLANLEWLSHFVEDSFTEYSGPNLTG<br>TPTEKPAWLTGDRKHPVTAVTEETCFKSPVPAKARSKRNRNGLKV<br>WSLGSSSSSGPSSSGSTSSSSSGPSSPWFSGAELLEPVVTSERPPFPK<br>KHKKRSAESVFSGELQQLQPQRKCSHCGVQKTPQWRAGPMGAK<br>TLCNACGVRYKSGRLLPEYRPACSPTFSSELHSNHHRKVIEMRRK<br>KEPTSDNETGLNQLVQSPQAVPSF
AtGATA6	MESVELTLKNSNMKDKTLTGGAQNGDDFSVDDLLDFSKEEEDDD<br>VLVEDEAELKVQRKRGVSDENTLHRSNDFSTADFHTSGLSVPMD<br>DIAELEWLSNFVDDSSFTPYSAPTNKPVWLTGNRRHLVQPVKEET<br>CFKSQHPAVKTRPKRARTGVRVWSHGSQSLTDSSSSSTTSSSSSPRP

	SSPLWLASGQFLDEPMTKTQKKKKVWKNAGQTQTQTQTQTRQC<br>GHCGVQKTPQWRAGPLGAKTLCNACGVRYKSGRLLPEYRPACSP<br>TFSSELHSNHHSKVIEMRRKKETSDGAEETGLNQPVQTVQVVSSF
AtGATA7	MECVEAFLGDFSVDDLLDLSNADTSLESSSSQRKEDEQEREKFKS<br>FSDQSTRLSPPEDLLSFPGDAPVGDLEDLEWLSNFVEDSFSESYISS<br>DFPVNPVASVEVRRQCVPVKPRSKRRRTNGRIWSMESPSPLLSTAV<br>ARRKKRGRQKVDASYGGVVQQQQLRRCCSHCGVQKTPQWRMG<br>PLGAKTLCNACGVRFKSGRLLPEYRPACSPTFTNEIHSNSHRKVLE<br>LRLMKVADPARV
AtGATA8	MIGTSFPEDLDCGNFFDNMDDLMDFPGGDIDVGFGIGDSDSFPTI<br>WTTHHDTWPAASDPLFSSNTNSDSSPELYVPFEDIVKVERPPSFVE<br>ETLVEKKEDSFSTNTDSSSSHSQFRSSSPVSVLESSSSSSQTTNTTSL<br>VLPGKHGRPRTKRPRPPVQDKDRVKDNVCGGDSRLIIRIPKQFLSD<br>HNKMINKKKKKKAKITSSSSSSGIDLEVNGNNVDSYSSEQYPLRK<br>CMHCEVTKTPQWRLGPMGPKTLCNACGVRYKSGRLFPEYRPAAS<br>PTFTPALHSNSHKKVAEMRNKRCSDGSYITEENDLQGLIPNNAYIG<br>VD
AtGATA9	MEKIAPELFLVAGNPDSFVVDDLLDFSNDDGEVDDGLNTLPDSST<br>LSTGTLTDSSNSSSLFTDGTGFSDLYIPNDDIAELEWLSNFVEESFA<br>GEDQDKLHLFSGLKNPQTTGSTLTHLIKPEPELDHQFIDIDESNVAV<br>PAKARSKRSRSAASTWASRLLSLADSDETNPKKKQRRVKEQDFAG<br>DMDVDCGESGGGRRCLHCATEKTPQWRTGPMGPKTLCNACGVR<br>YKSGRLVPEYRPASSPTFVMARHSNSHRKVMELRRQKEMRDEHL<br>LSQLRCENLLMDIRSNGEDFLMHNNTNHVAPDFRHLI
AtGATA10	MNWLPEAEAEEHLKGILSGDFFDGLTNHLDCPLEDIDSTNGEGDW<br>VARFQDLEPPPLDMFPALPSDLTSCPKGAARVRIPNNMIPALKQSCS<br>SEALSGINSTPHQSSAPPDIKVSYLFQSLTPVSVLENSYGSLSTQNS<br>GSQRLAFPVKGMRSKRRRPTTVRLSYLFPFEPRKSTPGESVTEGY<br>YSSEQHAKKKRKIHLITHTESSTLESSKSDGIVRICTHCETITTPQW<br>RQGPSGPKTLCNACGVRFKSGRLVPEYRPASSPTFIPSVHSNSHRKI<br>IEMRKKDDEFDTSMIRSDIQKVKQGRKKMV
AtGATA11	MNWLPEAEAEDDFKGLLSGDFFDDLINHLDVPLDDIDTTNGEGD<br>WVDRFQDLEPPPMDMFPTLPSDLTSCGSGMAKAPRVDIQRNIPAL<br>KQSYSSEALSSTLHQSSAPPEIKVSKLFQSLSPVSVLENSYGSLSTH<br>NNGSQRLAFPVKGMRSKRKRPTTLRLSYLFPSEPRKPEKSTPGKPE<br>SECYFSSEQHAKKKRKIHLTTRTVSSTLEASNSDGIVRKCTHCETT<br>KTPQWREGPSGPKTLCNACGVRFRSGRLVPEYRPASSPTFIPAVHS<br>NSHRKIIEMRRKDDEQFDSSMIRAVISRG

AtGATA12	MEDEAHEFFHTSDFAVDDLLVDFSNDDDEENDVVADSTTTTTITDS SNFSAADLPSFHGDVQDGTSFSGDLCIPSDDLADELEWLSNIVDES LSPEDVHKLELISGFKSRPDPKSDTGSPENPNSSSPIFTTDVSVPAK ARSKRSRAAACNWASRGLLKETFYDSPFTGETILSSQQHLSPPTSP PLLMAPLGKKQAVDGGHRRKKDVSSPESGGAEERRCLHCATDKT PQWRTGPMGPKTLCNACGVRYKSGRLVPEYRPAASPTFVLAKHS NSHRKVMELRRQKEMSRAHHEFIHHHHGTDTAMIFDVSSDGDDY LIHHNVGPDFRQLI
AtGATA13	MNNDLWLPEEDFKGLPDNFLDNLVDPTNDVSVEDIETGDDEGDW DAKFQKLVPPPLDELMSLSYEFTCNGQRVQVQKHVPILKQSSSSE VFSTVDNSPPNVKVSKLLQSLSPVSVLKNTNGSGSPQNPNGDQKL AFLVKGIRSKRKRPTLLRVTFLKSFLLEMSQQFAPDESESSEISALK KRKKNKSRRLKCTHCETTTTPQWREGPNGRKTLCNACGIRFRSG RLVLEYRPAASPTFIPTVHSNLHKKIIYMRMKDNDQFDTRKIRAET SGPETRSRLRNFGRPMSYGQ
AtGATA14	MSGREDEEEDLGTAMQKIPIPVNVFDKEPMDLDTVFGFADGVREII EDSNLLLEESREFDTNDSKPSRNFSNLPTATRGRLHAPKRSGNKRG RQKRLSFKSPSDLFDSKFGITDKSCSHCGTRKTPLWREGPRGAGTL CNACGMRYRTGRLLPEYRPASSPDFKPNVHSNFHRKVMEIRRERK SSPPNSFGFSESYHSTRKLGF
AtGATA15	MLDPTEKVIDSESMESKLTSVDAIEEHSSSSSNEAISNEKKSCAICG TSKTPLWRGGPAGPKSLCNACGIRNRKKRRTLISNRSEDKKKKSH NRNPKFGDSLKQRLMELGREVMMQRSTAENQRRNKLGEEEQAA VLLMALSYASSVYA
AtGATA16	MLDHSEKVLLVDSETMKTRAEDMIEQNNTSVNDKKKTCADCGTS KTPLWRGGPVGPKSLCNACGIRNRKKRRGGTEDNKKLKKSSSGG GNRKFGESLKQSLMDLGIRKRSTVEKQRQKLGEEEQAAVLLMAL SYGSVYA
AtGATA17	MSEGSEDTKTKLDSAGELSDVDNENCSSSGSGGGSSSGDTKRTCV DCGTIRTPLWRGGPAGPKSLCNACGIKSRKKRQAALGMRSEEKKK NRKSNCNNDLNLDHRNAKKYKINIVDDGKIDIDDDPKICNNKRSS SSSSNKGVSKFLDLGFKVPVMKRSAVEKKRLWRKLGEEERAAVL LMALSCSSVYA
AtGATA18	MMQTPYTTSTQGQYCHSCGMFHHHSQSCCYNNNNNSNAGSYSM VFSMQNGGVFEQNGEDYHHSSSLVDCTLSLGTPSTRLCEEDEKRR RSTSSGASSCISNFWDLIHTKNNNSKTAPYNNVPSFSANKPSRGCS GGGGGGGGGGGGDSLLARRCANCDTTSTPLWRNGPRGPKSLCN ACGIRFKKEERRTTAATGNTVVGAAPVQTDQYGHHNSGYNNYHA

	ATNNNNNNGTPWAHHHSTQRVPCNYPANEIRFMDDYGSGVANNV<br>ESDGAHGGVPFLSWRLNVADRASLVHDFTR
AtGATA19	MGFSMFFSPENDVSHHSSPYASVDCTLSLGTPSTRLCNEDDERRFS<br>SHTSDTIGWDFLNGSKKGGGGGGHNLLARRCANCDTTSTPLWRN<br>GPRGPKSLCNACGIRFKKEERRASTARNSTSGGGSTAAGVPTLDH<br>QASANYYYNNNNQYASSSPWHHQHNTQRVPYYSPANNEYSYVD<br>DVRVVDHDVTTDPFLSWRLNVADRTGLVHDFTM
AtGATA20	MMGYQTNSNFSMFFSSENDDQNHHNYDPYNNFSSSTSVDCTLSL<br>GTPSTRLDDHHRFSSANSNNISGDFYIHGGNAKTSSYKKGGVAHS<br>LPRRCASCDTTSTPLWRNGPKGPKSLCNACGIRFKKEERRATARN<br>LTISGGGSSAAEVPVENSYNGGGNYYSHHHHHYASSSPSWAHQNT<br>QRVPYFSPVPEMEYPYVDNVTASSFMSWN
AtGATA21	MDSNFHYSIDLNEDQNHHEQPFFYPLGSSSSLHHHHHHHHHQVPS<br>NSSSSSSSISSLSSYLPFLINSQEDQHVAYNNTYHADHLHLSQPLKA<br>KMFVANGGSSACDHMVPKKETRLKLTIRKKDHEDQPHPLHQNPT<br>KPDSDSDKWLMSPKMRLIKKTITNNKQLIDQTNNNNHKESDHYP<br>LNHKTNFDEDHHEDLNFKNVLTRKTTAATTENRYNTINENGYSNN<br>NGVIRVCSDCNTTKTPLWRSGPRGPKSLCNACGIRQRKARRAAM<br>AAAAAAGDQEVAVAPRVQQLPLKKKLQNKKKRSNGGEKYNHSPP<br>MVAKAKKCKIKEEEEKEMEAETVAGDSEISKSTTSSNSSISSNKFCF<br>DDLTIMLSKSSAYQQVFPQDEKEAAVLLMALSYGMVHG
AtGATA22	MGSNFHYTIDLNEDQNHQPFFASLGSSLHHHLQQQQQQQHFHH<br>QASSNPSSLMSPSLSYFPFLINSRQDQVYVGYNNNTFHDVLDTHIS<br>QPLETKNFVSDGGSSSSDQMVPKKETRLKLTIKKKDNHQDQTDLP<br>QSPIKDMTGTNSLKWISSKVRLMKKKKAIITTSDSSKQHTNNDQS<br>SNLSNSERQNGYNNDCVIRICSDCNTTKTPLWRSGPRGPKSLCNA<br>CGIRQRKARRAAMATATATAVSGVSPPVMKKKMQNKNKISNGVY<br>KILSPLPLKVNTCKRMITLEETALAEDLETQSNSTMLSSSDNIYFDD<br>LALLLSKSSAYQQVFPQDEKEAAILLMALSHGMVHG
AtGATA23	MDPRKLLSCSSSYVSVRMKEEKGTIRCCSECKTTKTPMWRGGPT<br>GPKSLCNACGIRHRKQRRSELLGIHIIRSHKSLASKKINLLSSSHGG<br>VAVKKRRSLKEEEQAALCLLLLSCSSVLA
AtGATA24	MDDLHGRNGRMHIGVAQNPMHVQYEDHGLHHIDNENSMMDDH<br>ADGGMDEGVETDIPSHPGNSADNRGEVVDRGIENGDQLTLSFQG<br>QVYVFDRVSPEKVQAVLLLLGGREVPHTLPTTLGSPHQNNRGLSG<br>TPQRLSVPQRLASLLRFREKRKGRNFDKTIRYTVRKEVALRMQRK<br>KGQFTSAKSSNDDSGSTGSDWGSNQSWAVEGTETQKPEVLCRHC<br>GTSEKSTPMMRRGPDGPRTLCNACGLMWANKGTLRDLSKVPPPQ

	TPQHLSLNKNEDANLEADQMMEVTGDISNTQ
AtGATA25	MFGRHSIIPNNQIGTASASAGEDHVSASATSGHIPYDDMEEIPHPDS<br>IYGAASDLIPDGSQLVAHRSDGSELLVSRPPEGANQLTISFRGQVYV<br>FDAVGADKVDAVLSLLGGSTELAPGPQVMELAQQQNHMPVVEY<br>QSRCSLPQRAQSLDRFRKKRNARCFEKKVRYGVRQEVALRMARN<br>KGQFTSSKMTDGAYNSGTDQDSAQDDAHPEISCTHCGISSKCTPM<br>MRRGPSGPRTLCNACGLFWANRGTLRDLSKKTEENQLALMKPDD<br>GGSVADAANNLNTEAASVEEHTSMVSLANGDNSNLLGDH
AtGATA26	MGKQGPCYHCGVTNTPLWRNGPPEKPVLCNACGSRWRTKGTLV<br>NYTPLHARADGDENDDHHRFQRMKSISLGNKNKEIKMLKRKAIQ<br>ENIIIKRPVFEFSYGLKAAVIEEDASNRSSSGSAVSNSESCAQFSSAD<br>GSPSQSNAWDTTVPCKRRTCVGRPKSSSVEKLTKDLYNILQEQQSS<br>CLSVSSEEDLLFENEMSMVSVEIGHGSVLMKNPHSFAREEESEASS<br>LSSIENKSSISDAYSHSVKRVEIGAVRGSYYGGQTIKQEQFKRTKSQ<br>TERVHVLGSHGSPLCSIDLKDVFNFDEFIEQFTEEEQKKLMNLLPQI<br>DSDDLPHSLRMMFESAQFKDNFSLFQQLIADGVFDVSSSSGAKLE<br>EIRTFKKLALTDFNKSRLVESYNLLKEREKGTGDSVTTTSKSSIPNV<br>PKNIVTIKRRYENQIQVKSESRGLMRSPKRVMKMKASHETENNVS<br>CFRPRSLASVFAQEGGSAVFSYEGNCSSDQDLLLLDLPSNGSFPQA<br>ELLHQL
AtGATA27	MGKQGPCYHCGVTSTPLWRNGPPEKPVLCNACGSRWRTKGSLVN<br>YTPLHARAEGDETEIEDHRTQTVMIKGMSLNKKIPKRKPYQENFT<br>VKRANLEFHTGFKRKALDEEASNRSSSGSVVSNSESCAQSNAWDS<br>TFPCKRRTCVGRPKAASSVEKLTKDLYTILQEQQSSCLSGTSEEDL<br>LFENETPMLLGHGSVLMRDPHSGAREEESEASSLLVESSKSSSVHS<br>VKFGGKAMKQEQVKRSKSQVLGRHSSLLCSIDLKDVFNFDEFIEN<br>FTEEEQQKLMKLLPQVDSVDRPDSLRSMFESSQFKENLSLFQQLV<br>ADGVFETNSSYAKLEDIKTLAKLALSDPNKSHLLESYYMLKRREIE<br>DCVTTTSRVSSLSPSNNNSLVTIERPCESLNQNFSETRGVMRSPKE<br>VMKIRSKHTEENLENSVSSFKPVSCGGPLVFSYEDNDISDQDLLLD<br>VPSNGSFPQAELLNMI
AtGATA28	MDDLHGSNARMHIREAQDPMHVQFEHHALHHIHNGSGMVDDQ<br>ADDGNAGGMSEGVETDIPSHPGNVTDNRGEVVDRGSEQGDQLTL<br>SFQGQVYVFDSVLPEKVQAVLLLLGGRELPQAAPPGLGSPHQNNR<br>VSSLPGTPQRFSIPQRLASLVRFREKRKGRNFDKKIRYTVRKEVAL<br>RMQRNKGQFTSAKSNNDEAASAGSSWGSNQTWAIESSEAQHQEI<br>SCRHCGIGEKSTPMMRRGPAGPRTLCNACGLMWANKGAFRDLSK<br>ASPQTAQNLPLNKNEDANLETDHQIMITVANDISNSQ
AtGATA29	MEPELDLTLKLGLPNSTVETHLTLSPPTTTTDQGTNVVDGGEVINH

	RRGLLGDDEVIHNEPTRNNVEFNIRIYNYVFQQFVGAPNTLNFAPY<br>PMPPSPAPAPETPPVSDEYVLIDVPARRARRNNSTVMTNSWKENA<br>TPKRIRGCGGFCGGRIEGMKKCTNMNCNALNTPMWRRGPLGPKS<br>LCNACGIKFRKEEERKAKRNVVIVLDD
AtGATA30	MSMTEETKTTKLESAGDSSDVDNGNCSSSGSGGDTKKTCVDCGT<br>SRTPLWRGGPAGPKSLCNACGIKSRKKRQAALGIRQDDIKIKSKSN<br>NNLGLESRNVKTGKGEPVNVKIAKCEPGIVKIAKGEPGNVKNKIK<br>RDPENSSSSNNNKKNVKRVGRFLDFGFKVPAMKRSAVEKKRLWR<br>KLGEEERAAVLLMALSCG
